# Associations of clinical parameter‐based accelerated aging, genetic predisposition with risk of chronic kidney disease and associated life expectancy: A prospective cohort study

**DOI:** 10.1111/acel.14453

**Published:** 2024-12-24

**Authors:** Gang Zheng, Qing Chang, Yixiao Zhang, Yashu Liu, Chao Ji, Honghao Yang, Liangkai Chen, Yang Xia, Yuhong Zhao

**Affiliations:** ^1^ Department of Clinical Epidemiology Shengjing Hospital of China Medical University, China Medical University Shenyang China; ^2^ Key Laboratory of Precision Medical Research on Major Chronic Disease Shenyang China; ^3^ Department of Urology Surgery Shengjing Hospital of China Medical University, China Medical University Shenyang China; ^4^ Department of General Surgery Shengjing Hospital of China Medical University, China Medical University Shenyang China; ^5^ Department of Nutrition and Food Hygiene, Hubei Key Laboratory of Food Nutrition and Safety, School of Public Health, Tongji Medical College Huazhong University of Science and Technology Wuhan China

**Keywords:** biological aging, chronic kidney disease, genetic predisposition, life expectancy

## Abstract

Little evidence exists regarding the associations between clinical parameter‐based biological aging and the incidence and outcome of chronic kidney disease (CKD). Thus, we aimed to assess the associations between biological aging, genetic risk, and the risk of CKD, as well as investigate the impact of accelerated biological aging on life expectancy. 281,363 participants free of kidney diseases from the UK Biobank were included in this prospective study. Biological age was measured from clinical traits using the KDM‐BA and PhenoAge algorithms, and the discrepancies from chronological age were defined as biological age accelerations. A polygenic score was calculated to indicate the genetic predisposition of the estimated glomerular filtration rate (eGFR). A cause‐specific competing risk model was used to estimate hazard ratios (HRs) and the corresponding confidence intervals (CIs) of incident CKD. We found that individuals with more pronounced accelerations in biological age exhibited an elevated risk of developing CKD (HR_Quartile 4 vs. Quartile 1_, 1.90; 95% CI, 1.77–2.05 for KDM‐BA acceleration and HR_Quartile 4 vs. Quartile 1_, 2.79; 95% CI, 2.58–3.01 for PhenoAge acceleration), with nonlinear relationships. Notably, there were positive additive interactions between biological aging and genetic risk on CKD risk. Among the CKD population, accelerated biological aging was associated with a further decline in life expectancy. Advanced biological aging may potentially increase the vulnerability to developing CKD in individuals aged midlife and beyond, particularly among those with high genetic risk for abnormal kidney function, and could reduce the life expectancy of CKD patients.

## Graphical abstract

By calculating clinical parameter‐based biological age for 281,363 adults from the UK Biobank study, we prospectively found that individuals with more pronounced accelerations in biological age exhibited an elevated risk of developing CKD, particularly among those with high genetic risk for abnormal kidney function. Moreover, accelerated biological aging was associated with a further decline in life expectancy among the CKD population. Under the circumstances of population aging, our findings highlighted the need for interventions targeting biological aging to mitigate CKD‐related health impacts.

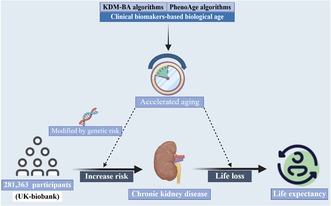

**Abbreviations**
AA: age acceleration
AP: attributable proportion due to interaction
BMI: body mass index
CI: confidence interval
CKD: chronic kidney disease
CVD: cardiovascular diseases
eGFR: estimated glomerular filtration rate
FEV1: forced expiratory volume in one second
HR: hazards ratio
hs‐CRP: high‐sensitive C‐reactive protein
KDM‐BA: Klemera‐Doubal method Biological Age
LDL‐C: low‐density lipoprotein cholesterol
PGS: polygenic score
RERI: relative excess risk due to interaction
SD: standard deviation
TDI: Townsend deprivation index
TG: triglycerides
UACR: urinary albumin‐to‐creatinine ratio

## 1 INTRODUCTION

Chronic kidney disease (CKD), which affected nearly 800 million individuals in 2019, imposes a substantial burden of premature mortality and health loss, posing a significant public health concern globally (“Global burden of 369 diseases and injuries in 204 countries and territories, 1990–2019: a systematic analysis for the Global Burden of Disease Study 2019,” 2020). Consequently, identifying risk factors and vulnerability mechanisms to CKD is a public health priority, essential for unraveling biological pathways that hold potential as therapeutic targets.

CKD has been characterized as a model of “accelerated aging”, and exhibits a high prevalence within the elderly population (Levey et al., 2020; Ortiz et al., 2023). A trans‐ethnic meta‐analysis of seven population‐based studies revealed that DNA methylation‐based indicators of accelerated aging in blood were associated with decreased kidney function (Matías‐García et al., 2021). Furthermore, a recent Mendelian randomization study utilizing UK‐Biobank data has uncovered a causal relationship between biological aging measured by telomere attrition and renal function impairment (Park et al., 2021). Nevertheless, aging represents an intricate biological process characterized by a gradual erosion of cellular, tissular, and organ integrity and resilience capacity (Kennedy et al., 2014). Thus, ideal measurements of biological aging should be derived from comprehensive algorithms that integrate information from multiple biological systems across epigenetic, proteomic, metabolomic, and other molecular levels, rather than relying solely on individual biomarkers (Ferrucci et al., 2020; Khan et al., 2017). Among these algorithms, those incorporating data from conventional clinical indicators have shown superior accuracy in predicting disease, disability, and mortality (Belsky et al., 2018; Li et al., 2020). However, no work has applied clinical parameter‐based biological age algorithms to explore the associations of accelerated processes in biological aging with incident and mortality risk of CKD, which may provide a novel potential target for risk assessment and intervention on CKD. Furthermore, genetic factors may significantly affect kidney function (Thio et al., 2018). While previous studies have explored the influence of genetic factors on the associations of intrinsic physiological status with CKD risk (Yang et al., 2024), the potential interaction between accelerated biological aging and genetic susceptibility to CKD development remains unknown.

In this study, we computed biological age values for participants based on blood chemistries collected from UK Biobank participants at their baseline assessment, using the published and validated Klemera‐Doubal method Biological Age (KDM‐BA) and PhenoAge algorithms (Graf et al., 2022; Liu et al., 2018). We aimed to evaluate the associations between the two biological ages and the risk of incident CKD as well as the potential gene–environment interactions between genetic predisposition and biological aging status. Additionally, we also assessed how accelerated biological aging affected life expectancy among people with CKD.

## 2 METHODS

### 2.1 Study design and population

The UK Biobank is a prospective population‐based cohort study comprising more than 500,000 individuals aged between 40 and 69 years residing in the United Kingdom with the time of recruitment from 2006 to 2010. Participants were requested to complete a touch screen survey, participate in an in‐person interview conducted by a nurse, and undergo various physical assessments. Participants were also required to provide biological samples for genotype and biomarker analysis. Detailed information regarding the design and study population of UK Biobank can be found on their official website (https://www.ukbiobank.ac.uk/) and in previous studies (Fry et al., 2017; Sudlow et al., 2015). All participants voluntarily provided written consent for their involvement in the study, and the research has received approval from the North West Multi‐Center Research Ethics Committee (Manchester, U.K.).

The present analysis utilized data from 502,617 participants enrolled in the UK Biobank. Of 409,999 participants with available data on the KDM‐BA and the PhenoAge algorithms, we excluded those with prevalent CKD at baseline [diagnosed CKD using codes, estimated glomerular filtration rate (eGFR) <60 mL/min/1.73 m^2^, urinary albumin‐to‐creatinine ratio (UACR) ≥30 mg/g, or self‐reported CKD] (*n* = 27,988), those with missing data on potential covariates (*n* = 76,964), or those with pre‐existing malignancy (*n* = 23,684). This resulted in a total of 281,363 participants included in the final analysis (Figure S1).

### 2.2 Evaluation of biological ages and age accelerations

We utilized well‐validated KDM‐BA and PhenoAge algorithms to assess biological age based on available blood chemistry data from the UK Biobank (Levine, 2013; Liu et al., 2018). Details of these biological age algorithms and corresponding codes for UK Biobank data fields have been described previously (Gao et al., 2023). Briefly, the KDM‐BA algorithm considered forced expiratory volume in one second (FEV_1_), systolic blood pressure, and seven specific blood chemistry parameters (albumin, alkaline phosphatase, blood urea nitrogen, creatinine, C‐reactive protein, glycated hemoglobin, and total cholesterol). The KDM‐BA is derived from a series of regressions of the above individual biomarkers on chronological age in a reference population (Levine, 2013). The original PhenoAge algorithm was constructed from elastic‐net Gompertz regression of mortality on 42 biomarkers in the NHANES III (Liu et al., 2018). This analysis selected nine blood chemistries including four that overlapped with KDM‐BA (albumin, alkaline phosphatase, creatinine, and C‐reactive protein) as well as glucose levels, mean cell volume, red cell distribution width, white blood cell count, and lymphocyte proportion. Included biomarkers in biological age algorithms are reported in Table 1. The R package “BioAge” (https://github.com/dayoonkwon/BioAge) was employed to compute biological age values (Kwon & Belsky, 2021).

**TABLE 1:** Baseline characteristics according to lowest and highest quartiles of biological age accelerations in 281,363 participants.

Characteristics	Total *N* = 281,363	KDM‐BA acceleration		PhenoAge acceleration	
		Quartile 1	Quartile 4	Quartile 1	Quartile 4
Sociodemographic factors					
Age at recruitment (years)	55.8 (8.1)	56.6 (8.1)	55.6 (7.9)	55.2 (7.9)	56.3 (8.2)
Female sex, *n* (%)	143,250 (50.9)	4088 (5.8)	58,347 (83)	43,999 (62.6)	31,032 (44.1)
White ethnicity, *n* (%)	266,707 (94.8)	68,115 (96.8)	64,453 (91.6)	67,204 (95.5)	65,597 (93.3)
Townsend deprivation index	−1.42 (3.02)	−1.64 (2.93)	−1.12 (3.16)	−1.73 (2.85)	−0.92 (3.24)
College or university degree, *n* (%)	32,844 (11.7)	9616 (13.7)	6622 (9.4)	9428 (13.4)	6861 (9.75)
Income >52,000 EUR, *n* (%)	65,822 (23.4)	19,870 (28.3)	12,094 (17.2)	19,125 (27.2)	12,823 (18.2)
Lifestyle information					
BMI (kg/m^2^)	27.23 (4.6)	26.5 (3.65)	28.65 (5.37)	25.21 (3.52)	29.27 (5.45)
Current smoker, *n* (%)	28,760 (10.2)	6501 (9.2)	8143 (11.6)	3675 (5.2)	12,806 (18.2)
Current alcohol drinker, *n* (%)	261,251 (92.9)	66,615 (94.7)	63,471 (90.2)	66,035 (93.9)	63,910 (90.9)
Physical activity (MET min/wk)	2676 (2731)	2803 (2840)	2517 (2619)	2743 (2629)	2544 (2814)
eGFR (mL/min/1.73 m^2^)	92.29 (11.82)	92.69 (11.09)	91.08 (12.57)	95.89 (10.24)	89.41 (12.92)
UACR (mg/g)	9.88 (6.18)	8.73 (5.85)	10.93 (6.39)	10.18 (6.2)	10 (6.31)
Comorbidity					
Cardiovascular disease, *n* (%)	11,576 (4.1)	3013 (4.3)	3202 (4.6)	2396 (3.4)	5430 (7.7)
Hypertension, *n* (%)	67,589 (24.0)	15,676 (22.3)	21,051 (29.9)	12,271 (17.5)	22,251 (31.6)
Diabetes, *n* (%)	14,520 (5.2)	3666 (5.2)	5112 (7.3)	1188 (1.7)	6066 (8.6)
Components of biological ages					
FEV_1_ (L)^a^	2.8 (0.79)	3.66 (0.67)	2.15 (0.53)	2.83 (0.78)	2.7 (0.78)
SBP (mm Hg)^a^	137.1 (18.2)	134.7 (15.5)	144.4 (19.2)	134.0 (18.1)	139.2 (18.1)
Total Cholesterol (mg/dL)^a^	220.1 (43.4)	208.8 (41.1)	236.7 (45.4)	222.6 (42.2)	214.8 (44.7)
Glycated hemoglobin (%)^a^	5.42 (0.56)	5.34 (0.47)	5.54 (0.7)	5.27 (0.36)	5.64 (0.82)
Albumin (g/dL)^a^, ^b^	4.53 (0.26)	4.59 (0.25)	4.49 (0.26)	4.62 (0.25)	4.43 (0.26)
Creatinine (mg/dL)^a^, ^b^	0.81 (0.15)	0.89 (0.13)	0.76 (0.14)	0.75 (0.13)	0.85 (0.16)
C‐reactive protein (mg/dL)^a^, ^b^	2.39 (3.99)	1.17 (1.91)	4.09 (5.75)	0.75 (0.7)	5.25 (6.67)
Alkaline phosphatase (U/L)^a^, ^b^	82.17 (24.74)	76.71 (19.24)	91.77 (31.05)	74.95 (19.5)	91.51 (31.75)
Blood urea nitrogen (mg/dL)^a^	14.97 (3.48)	15.08 (3.32)	15.43 (3.64)	14.47 (3.23)	15.21 (3.77)
Lymphocyte (%)^b^	29.06 (7.29)	28.59 (7.2)	29.34 (7.46)	32.2 (7.03)	25.92 (7.19)
Mean cell volume (fL)^b^	82.82 (5.24)	82.95 (5.15)	82.62 (5.32)	81.37 (4.63)	84.15 (6.07)
Red cell distribution width (%)^b^	13.45 (0.94)	13.37 (0.79)	13.56 (1.04)	12.94 (0.53)	14.14 (1.3)
White blood cell count (1000 cells/uL)^b^	6.81 (1.76)	6.49 (1.64)	7.23 (1.87)	5.82 (1.23)	7.97 (2.07)
Serum glucose (mg/dL)^b^	91.31 (20.28)	90.15 (17.44)	93.51 (24.93)	86.82 (10.49)	98.58 (33.32)

*Note*: Numbers are presented as means (standard deviation) unless otherwise specified as numbers (%).

Abbreviations: BMI, body mass index; eGFR, estimated glomerular filtration rate; FEV_1_, forced expiratory volume in 1 s; MET, metabolic equivalent task; SBP, systolic blood pressure; UACR, urine albumin to creatinine ratio.

^a^ Components of KDM‐BA.

^b^ Components of PhenoAge.

Given that an elevated biological age relative to chronological age may indicate unhealthy aging processes (Earls et al., 2019), we employed the concept of “age acceleration (AA)” to quantitatively assess disparities between participants in biological aging, which was defined as the discrepancy between biological ages and chronological ages (biological ages minus chronological ages). This approach offers several advantages as it provides an individual‐level measure instead of a population‐level measure and does not assume a mean of zero within each population, unlike the residual‐based measure.

### 2.3 Polygenic score for kidney function

The genotyping process and quality control measures in the UK Biobank study have been extensively discussed elsewhere (Bycroft et al., 2018). A polygenic score (PGS) was calculated for kidney function using a weighted approach based on 263 single nucleotide polymorphisms, each exhibiting significant genome‐wide associations with eGFR levels (Table S1). Detailed information for the calculation of the PGS for eGFR is provided in Appendix S1. A higher PGS of eGFR indicates an increased genetic inclination toward elevated eGFR levels and a decreased genetic risk for abnormal renal function. We then classified participants into high genetic risk groups (below the median) and low genetic risk groups (above the median) for abnormal renal function based on the PGS of eGFR.

### 2.4 Covariates and outcomes

The questionnaire assessments of sociodemographic, dietary, and lifestyle factors of the UK Biobank cohort were conducted during baseline examinations between 2006 and 2010. Proficient staff meticulously collected anthropometric measurements as well as biological specimens from all study participants. Further information on the covariates used in the present study can be found in Appendix S1. The CKD case ascertainment in the UK Biobank cohort is also described detailedly in Appendix S1. Briefly, the incident CKD cases were determined by utilizing the International Classification of Diseases, 10th Revision (ICD‐10) codes (E10.2, E11.2, I12.x, I13.x, N03.x, N11.x, N18.x, T86.1, Z49.x, Z94.0, and Z99.2) present in primary care data, hospital inpatient data, and death register records as well as Office of Population Censuses and Surveys Classification of Interventions and Procedures‐version 4 (OPCS‐4) codes (L74.1–74.6, L74.8–74.9, M01.2–01.9, M02.3, M08.4, M17.2, M17.4, M17.8–17.9, X40.1–40.9, X41.1–41.2, X41.8–41.9, X42.1, and X42.8–42.9) from hospital inpatient data.

### 2.5 Statistical analysis

The summary measures were presented as mean [standard deviation (SD)] for continuous variables and as count (percentage) for categorical ones. Cumulative incidence curves for kidney outcome were derived using cumulative incidence functions, compared using Gray's test. The relations of baseline AAs with incident CKD were estimated using the cause‐specific competing risk model (hazards ratio [HR] and 95% confidence interval [CI]), treating deaths before CKD development as competing risks and censored. Tests for linear trend were performed using the median of each quartile of AAs as a continuous variable or per SD increase in AAs. We fitted a series of models adjusting for increasing numbers of covariates which were selected based on prior knowledge and epidemiological evidence (Bello et al., 2008; Burnier & Damianaki, 2023; Kelly et al., 2021; Levey et al., 2016; Nunes et al., 2014; Weiner et al., 2006): Model 1 was not adjusted; Model 2 was adjusted for age (years, continuous), sex (male, female), race (White, Asian, Black, Mixed), body mass index (BMI) (kg/m^2^, continuous), Townsend deprivation index (TDI) (continuous), smoking status (never, previous, current), alcohol consumption (daily or almost daily, 1–4 times a week, 1–3 times a month, never or special occasions only), education (college or university degree, high school, below), and physical activity (MET‐min/week, continuous); Model 3 was additionally adjusted for hypertension, cardiovascular diseases (CVD), and diabetes at baseline (yes or no). The proportional hazards assumption was tested by the Schoenfeld residuals test, and no significant deviation from proportionality in hazards over time was detected. The restricted cubic splines were used to flexibly model the associations between AAs and incident CKD risks, with the minimum Akaike information criterion used to choose optimal knots. To make effect sizes for the two AAs comparable, we standardized the AAs to continuous variables with per SD increase for analysis.

The combined effects of AAs and genetic risk were evaluated by generating dummy variables based on joint exposure to both factors, with the first 10 genetic principal components and genotyping batch further adjusted in the final model. The potential presence of additive interactions between AAs and genetic risk was investigated by calculating the relative excess risk due to interaction (RERI) and attributable proportion due to interaction (AP) (Li & Chambless, 2007). The 95% CIs of RERI and AP were generated by drawing 3000 bootstrap samples from the estimation dataset. To assess for multiplicative interaction, the cause‐specific competing risk models incorporated an interaction term for AAs and the PGS of eGFR, with statistical significance evaluated through the Wald test. Analyses were also stratified by the PGS category to assess for an effect modification between genetic risk and AAs.

We conducted proportional hazards survival analyses using the stpm2 command in Stata to evaluate the impact of biological aging on life expectancy. Given that chronic diseases tend to have higher prevalence rates and mortality data becomes more accessible and reliable after reaching the age of 45 (Chen et al., 2024), our findings primarily focus on elucidating how biological aging specifically influences mean life expectancy at age 45 among individuals with and without CKD. To estimate residual life expectancy, we calculated the area under the survival curve up to 100 years of age (Chudasama et al., 2019).

We further conducted subgroup analyses according to age (<60 or ≥60 years), gender (male or female), BMI (<25 or ≥25 kg/m^2^), physical activity (below or above the median value), TDI (below or above the median value), history of diabetes (yes or no), and history of hypertension (yes or no). *p* for multiplicative interactions between these covariates and AAs were calculated by entering a multiplication term into the original models. Several sensitivity analyses were performed to test the robustness of the findings by: (i) excluding individuals within the first 2 years of follow‐up to reduce the possibility of reverse causation; (ii) using the Fine and Gray's model to estimate the sub‐distribution HRs (95% CIs) of incident CKD; (iii) additionally adjusting for eGFR to mitigate the potential confounding effect of baseline renal function; (iv) additionally adjusting for cardiometabolic biomarkers (triglycerides [TG], low‐density lipoprotein cholesterol [LDL‐C], high‐sensitive C‐reactive protein [hs‐CRP]); (v) excluding individuals with history of CVD and diabetes.

A two‐tailed *p* < 0.05 was deemed statistically significant in all analyses. SAS version 9.4, R software version 4.2.0, and Stata version 17.0 were utilized for performing all statistical analyses.

## 3 RESULTS

### 3.1 Baseline characteristics of participants by biological AAs

Of 281,363 CKD‐free participants, 7945 (2.82%) developed CKD during a median follow‐up period of 12.3 years. Distributions of the baseline characteristics by the lowest and highest quartiles of AAs are shown in Table 1. The age (mean ± SD) of participants eligible for the analysis was 55.8 (8.1) years and most of them were white (94.8%). The proportion of females was 50.9%, and the mean TDI of the participants was −1.42 ± 3.02. About 24%, 5.2%, and 4.1% of participants were with hypertension, diabetes, and CVD, respectively. In general, individuals with advanced biological AAs were more likely to be less educated, lower‐paid, current smokers, and have higher levels of BMI. They also tended to have inferior renal function and more comorbidities at baseline. In addition, participants' biological ages were highly correlated with their chronological ages (Table S2).

### 3.2 Associations between biological age accelerations and incident CKD

**FIGURE 1:**
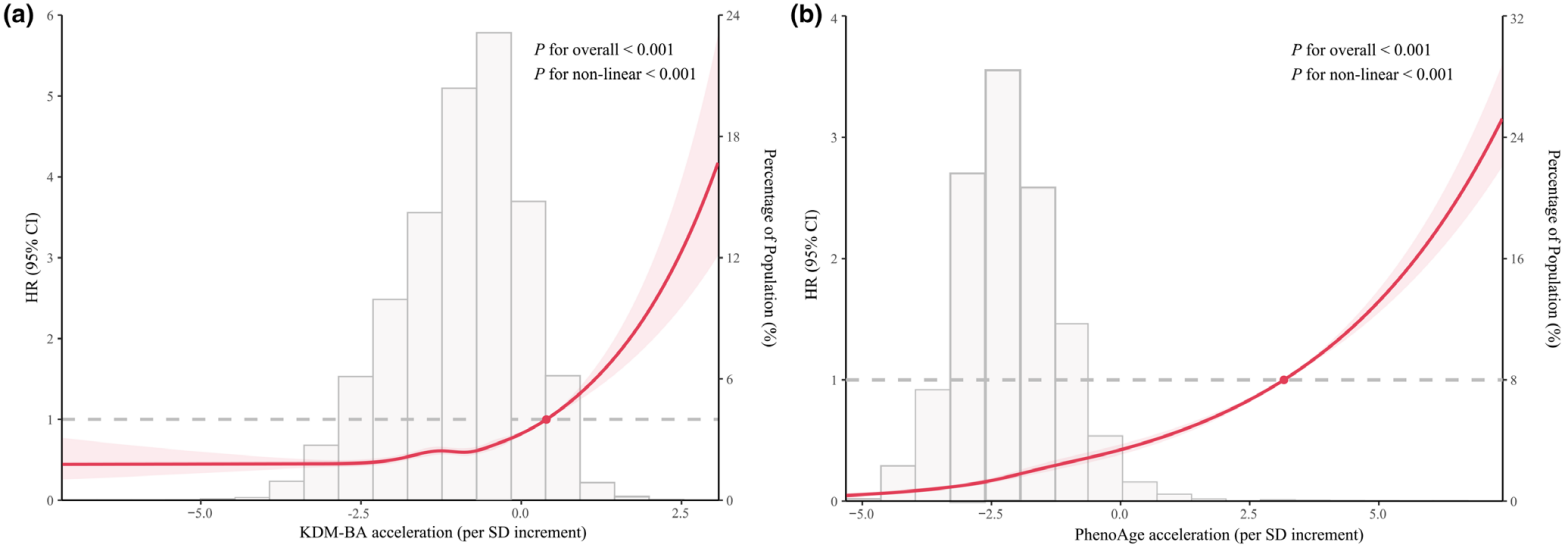
Cubic spline analysis for the associations of KDM‐BA acceleration (a) and PhenoAge acceleration (b) with incident CKD in 281,363 participants. CIs, confidence intervals; CKD, chronic kidney disease; HRs, hazard ratios; SD, standard deviation. HRs are indicated by red lines and 95% CIs by shaded areas. knots were selected based on the minimum Akaike information criterion. All models were adjusted for age (years, continuous), sex (male, female), race (White, Asian, Black, Mixed), BMI (kg/m^2^, continuous), Townsend deprivation index (continuous), smoking status (never, previous, current), alcohol consumption (daily or almost daily, 1–4 times a week, 1–3 times a month, never or special occasions only), education (college or university degree, high school, below), physical activity (MET‐min/week, continuous), and history of hypertension, cardiovascular disease, diabetes (yes, no).

The cumulative incidence curve for incident CKD showed that events were significantly higher in participants with advanced acceleration in biological age (Figure S2A,B). Moreover, the incidence rates of CKD across AAs quartile groups were also graded increased (Table 2). In the multivariable cause‐specific competing risk model adjusted for age, sex, race, BMI, TDI, education, smoking status, alcohol consumption, physical activity, and history of hypertension, cardiovascular disease, and diabetes, we found that participants in the highest quartile of KDM‐BA acceleration (HR, 1.90; 95% CI, 1.77–2.05; *p*‐trend <0.0001) and PhenoAge acceleration (HR, 2.79; 95% CI, 2.58–3.01; *p*‐trend <0.0001) were both associated with an increased risk of CKD, compared with those in the lowest quartiles (Table 2). Moreover, each SD increase in biological AAs (HR, 1.30; 95% CI, 1.26–1.34 for KDM‐BA and HR, 1.43; 95% CI, 1.41–1.46 for PhenoAge) was associated with a 30%–43% increase in the risk of incident CKD (Table 2). The restricted cubic spline analysis revealed non‐linear associations with CKD risk for both KDM‐BA acceleration and PhenoAge acceleration (*p*‐nonlinearity <0.001 for both AAs; the risk of CKD began relatively stable and then started to increase rapidly afterward with the increase of AAs) (Figure 1a,b).

**TABLE 2:** Associations (HRs and 95% CIs) between biological age accelerations at baseline and incident CKD in 281,363 participants^a^.

Biological ages	Cases (*n*, %)	Model 1	Model 2	Model 3
		HR (95% CI)	HR (95% CI)	HR (95% CI)
KDM‐BA acceleration (Quartiles)				
Q1	1711 (2.43%)	1.00 (Ref)	1.00 (Ref)	1.00 (Ref)
Q2	1811 (2.57%)	1.05 (0.98, 1.12)	1.21 (1.13, 1.30)	1.21 (1.13, 1.29)
Q3	1765 (2.51%)	1.02 (0.95, 1.09)	1.36 (1.26, 1.46)	1.35 (1.26, 1.46)
Q4	2658 (3.78%)	1.55 (1.46, 1.64)	1.91 (1.78, 2.06)	1.90 (1.77, 2.05)
*p*‐trend^b^		<0.0001	<0.0001	<0.0001
Continuous (per SD increase)		1.20 (1.17, 1.22)	1.30 (1.27, 1.34)	1.30 (1.26, 1.34)
PhenoAge acceleration (Quartiles)				
Q1	858 (1.22%)	1.00 (Ref)	1.00 (Ref)	1.00 (Ref)
Q2	1364 (1.94%)	1.61 (1.47, 1.75)	1.37 (1.26, 1.49)	1.38 (1.26, 1.50)
Q3	2031 (2.89%)	2.41 (2.23, 2.61)	1.82 (1.68, 1.98)	1.83 (1.69, 1.99)
Q4	3692 (5.25%)	4.52 (4.20, 4.87)	2.98 (2.75, 3.22)	2.79 (2.58, 3.01)
*p*‐trend^b^		<0.0001	<0.0001	<0.0001
Continuous (per SD increase)		1.61 (1.59, 1.63)	1.49 (1.46, 1.51)	1.43 (1.41, 1.46)

*Note*: Model 1 was not adjusted. Model 2: adjusted for age (years, continuous), sex (male, female), race (White, Asian, Black, Mixed), BMI (kg/m^2^, continuous), Townsend deprivation index (continuous), education (college or university degree, high school, below), smoking status (never, previous, current), alcohol consumption (daily or almost daily, 1–4 times a week, 1–3 times a month, never or special occasions only), and physical activity (MET‐min/week, continuous). Model 3: Model 2 + history of hypertension, cardiovascular disease, diabetes (yes, no).

Abbreviations: CIs, confidence intervals; CKD, chronic kidney disease; HRs, hazard ratios; SD, standard deviation.

^a^ HR and 95% CI were calculated using the cause‐specific competing risk model.

^b^ Test for trend based on variables containing the median value for each quartile.

### 3.3 Effect modification by genetic susceptibility

**FIGURE 2:**
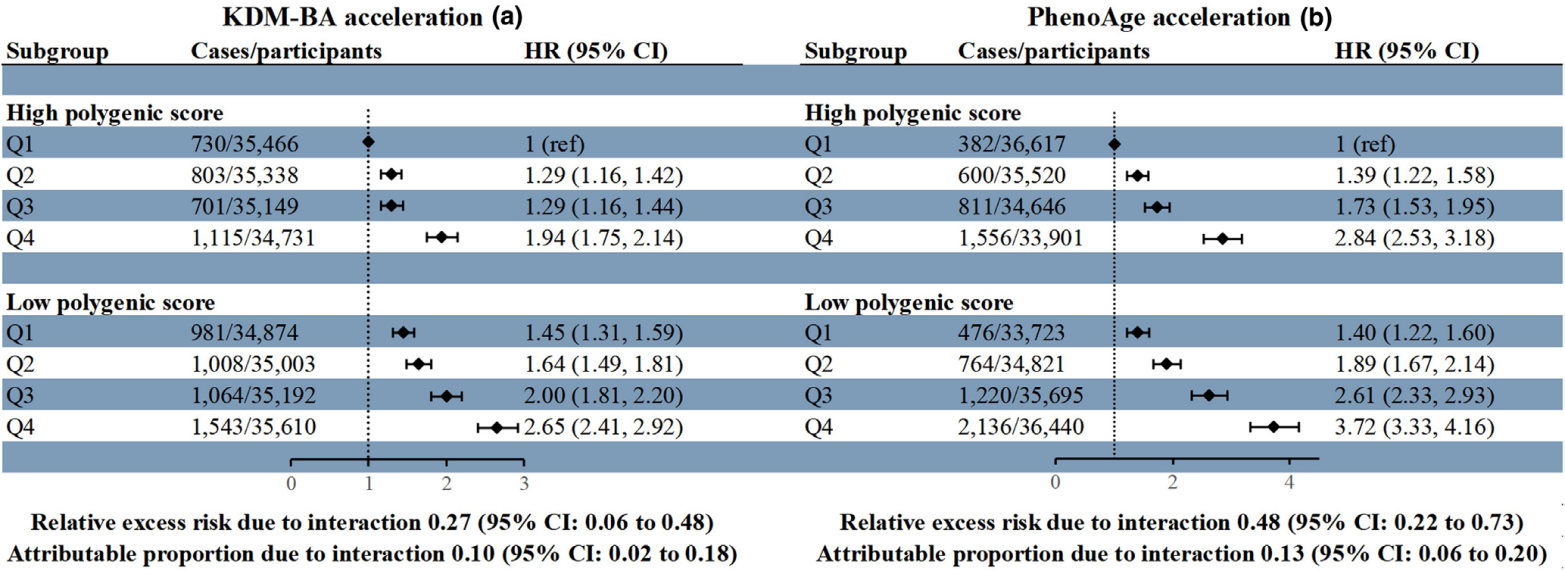
HRs and 95% CIs of incident CKD according to cross groups^a^ of KDM‐BA acceleration (a) and PhenoAge acceleration (b) with polygenic score for eGFR^b^ (*n* = 281,363). CIs, confidence intervals; CKD, chronic kidney disease; eGFR, estimated glomerular filtration rate; HRs, hazard ratios; Q, quintile; ref., reference group. HRs were calculated from cause‐specific competing risk models adjusted for age (years, continuous), sex (male, female), race (White, Asian, Black, Mixed), BMI (kg/m^2^, continuous), Townsend deprivation index (continuous), smoking status (never, previous, current), alcohol consumption (daily or almost daily, 1–4 times a week, 1–3 times a month, never or special occasions only), education (college or university degree, high school, below), physical activity (MET‐min/week, continuous), history of hypertension, cardiovascular disease, diabetes (yes, no), first 10 principal components of ancestry, and genotyping batch. Additive interaction was assessed by calculating the relative excess risk due to interaction and attributable proportion due to interaction between KDM‐BA acceleration or PhenoAge acceleration (Q4 vs. Q1) and polygenic score for eGFR (low vs. high). The statistical significance of the additive interaction was determined based on whether its confidence interval excluded 0. ^a^Reference group was participants with high polygenic score and the lowest quartile of KDM‐BA acceleration or PhenoAge acceleration. ^b^High polygenic score for eGFR indicates a lower genetic risk of abnormal renal function, with a low polygenic score indicating a higher genetic risk.

In the current study, a significant association was observed between the high PGS of eGFR and a reduced risk of incident CKD (HR, 0.71; 95% CI, 0.68–0.74; Table S3). We observed joint associations of genetic predisposition and biological AAs with CKD risk that behaved in a dose–response manner, with the highest risk of incident CKD for participants who had the highest levels of both genetic risk and biological AAs (HR, 2.65; 95% CI, 2.41–2.92 for KDM‐BA acceleration and HR, 3.72; 95% CI, 3.33–4.16 for PhenoAge acceleration). Notably, we observed positive additive interactions for older biological age with high genetic risk on incident CKD (Figure 2). The RERIs and APs were 0.27 (95% CI, 0.06–0.48) and 0.10 (95% CI, 0.02–0.18) for KDM‐BA acceleration, while the corresponding values for PhenoAge acceleration were 0.48 (95% CI, 0.22–0.73) and 0.13 (95% CI, 0.06–0.20). The observed additive interactions suggested that genetic susceptibility may significantly exacerbate the associations between biological AAs and incident CKD. In addition, participants with advanced acceleration in biological aging had an increased risk of CKD across all PGS groups (Figure S3).

### 3.4 Associations between biological age accelerations and life expectancy

**FIGURE 3:**
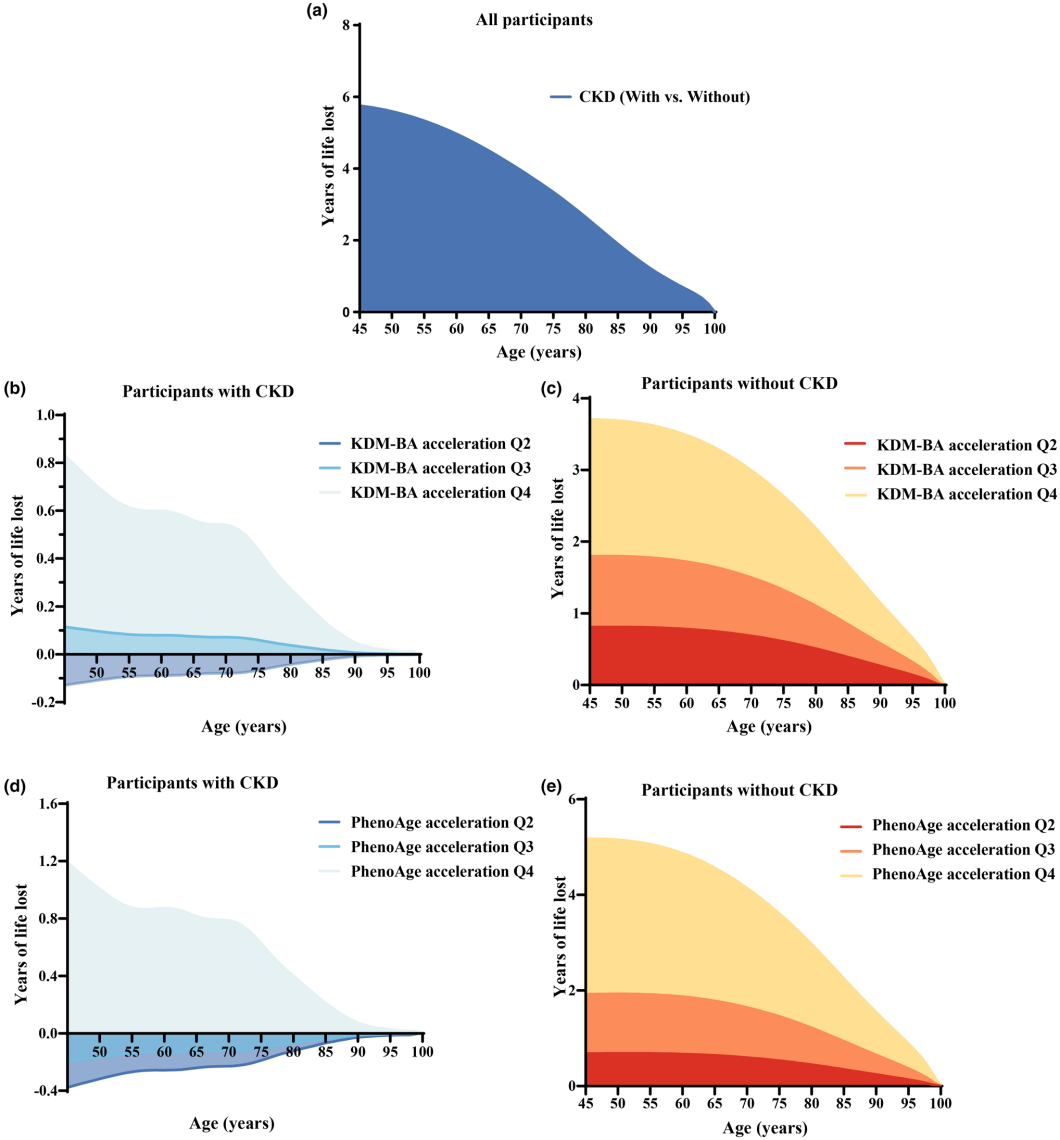
Associations of KDM‐BA acceleration and PhenoAge acceleration with life expectancy lost stratified by CKD status in 281,363 participants. CKD, chronic kidney disease. Years of life lost among participants with CKD compared with those without CKD (a). Years of life lost in other quartiles by KDM‐BA acceleration and PhenoAge acceleration compared with quartile 1 among people with CKD (b, d). Years of life lost in other quartiles by KDM‐BA acceleration and PhenoAge acceleration compared with quartile 1 among people without CKD (c, e).

The results of our life expectancy analysis revealed that individuals aged 45 years or older with CKD had a significantly shorter life expectancy compared to those without the disease (Figure 3a). Among participants at the age of 45 years, life expectancy was 32.5 years (95% CI, 32.0–33.0) in people with CKD and 38.3 (95% CI, 37.8–38.8) for those without the disease (Table S4). Accelerated biological aging was associated with reduced life expectancy among people at age 45 years with CKD as well as without CKD (Figure 3b–e). At age 45 years, people with CKD who were at the highest quartile of biological AAs had a loss of life expectancy ranging from 0.8 years (95% CI 0.3–1.4; KDM‐BA) to 1.2 years (0.5–1.9; PhenoAge) compared with those in the lowest quartile (Table S4). The pattern of results for biological AAs was similar among individuals who did not have CKD with a loss of life expectancy ranging from 3.7 years (95% CI, 3.3–4.1; KDMAge) to 5.2 years (95% CI, 4.7–5.6; PhenoAge) (Table S4). We also noted that at age 45 years, people with CKD and accelerated biological aging exhibited a significantly diminished life expectancy from 26.3 (95% CI, 25.8–26.9; PhenoAge) to 26.6 (95% CI, 26.0–27.2; KDMAge).

### 3.5 Subgroup and sensitivity analyses

The associations of both AAs with CKD did not significantly alter across major subgroups of participants defined by age, gender, BMI, TDI, physical activity, history of diabetes, and history of hypertension (Figure S4). However, we observed significant interactions between both AAs and chronological age, between PhenoAge acceleration and gender, and history of hypertension as well as between KDM‐BA acceleration and history of diabetes (*p* for interaction <0.05 for all). Notably, the results indicated that individuals under 60 years old experiencing biological aging acceleration had a heightened risk of CKD compared to older individuals. This finding underscored the importance of early screening and preventive measures on AAs for younger individuals to mitigate the risk of developing CKD. To validate our findings, we also performed several sensitivity analyses. The results remained consistent in separate sensitivity analyses that either excluded participants with less than 2 years of follow‐up (Table S5), utilized the Fine and Gray's model (Table S6), additionally adjusted for cardiometabolic biomarkers (TG, LDL‐C, and hs‐CRP) and eGFR (Table S7), or excluded participants with pre‐existing CVD and diabetes (Table S8).

## 4 DISCUSSION

In this large prospective cohort, we investigated the associations between biological ages derived from clinical biomarkers and incident CKD among middle‐aged and older adults. Our findings revealed that individuals with more pronounced accelerations in biological age exhibited an elevated risk of developing CKD over a median follow‐up period of 12.3 years. Notably, there were significant synergistic effects between biological AAs and genetic risk on CKD risk. We further observed that people with older biological age and high genetic risk had the highest risk of CKD. Furthermore, our results suggested that among people with CKD, accelerated biological aging was associated with a decreased life expectancy at 45 years or older. These findings suggest potential future strategies for assessing the risk of CKD in midlife and elderly individuals and the prospect of employing interventions targeting age‐related biological processes to mitigate CKD progression later in life.

There is accumulating evidence for a link between biological aging and renal function. A large trans‐ethnic meta‐analysis of up to 9688 individuals reported that DNA methylation AA estimated in whole blood exhibited significant associations with various kidney traits, including eGFR and CKD (Matías‐García et al., 2021). Recent Mendelian randomization studies supported bidirectional causal relationships between kidney function and epigenetic AA or telomere attrition (Pan et al., 2023; Park et al., 2021). However, most of these studies have focused on single biological markers that correlate with age, although metrics combining multiple biological measures can have stronger correlations with chronological age and are increasingly being investigated as potential predictors of morbidity and mortality (Holly et al., 2013; Levine, 2013; Peters et al., 2015). The current study is the first to demonstrate prospective associations between accelerated biological aging measured based on multiple blood biochemistry biomarkers and elevated CKD risk. Also, we noted response curves with different features of both KDM‐BA and PhenoAge accelerations with CKD risk. This discrepancy can be attributed to KDM‐biological age being more closely linked to the capacity and functionality of systems and organs, and PhenoAge tends to skew toward predicting human mortality risk (Hastings et al., 2019; Liu et al., 2018). Conclusively, our findings indicated that if these aging‐related biomarkers included in both KDM‐BA and PhenoAge measurements such as FEV_1_, systolic blood pressure, and other specific blood chemistry parameters can be modified, which could potentially be attained through adopting healthy lifestyles or future interventions, age‐related kidney function decline may not be inevitable or may even be preventable.

Existing gene–environment studies on CKD were primarily focused on multiplicative interaction, and interaction on the additive scale is often overlooked, despite its crucial role in determining biological interaction between risk factors and its implications for etiology. In the current study, we observed interactions on the additive scale between biological aging and genetic risk for abnormal kidney function, which suggested that biological aging and genetic risk may have a stronger joint effect on the risk of CKD rather than the sum of individual effects. Moreover, the observed additive interactions hold more significant implications for public health as they enable the identification of individuals who are more likely to derive benefits from targeted interventions on biological age (VanderWeele & Knol, 2014). Specifically, our study emphasizes the imperative of heightened attention toward individuals with elevated genetic risk regarding their biological aging status. Furthermore, interventions targeting the aging process, such as anti‐aging medication and health promotion, may yield greater benefits among individuals with an augmented genetic predisposition to CKD. Biological interaction occurs when two risk factors synergistically contribute to the same disease pathway (Ahlbom & Alfredsson, 2005). However, the exact mechanisms underlying the interplay between genetic susceptibility to kidney function and biological aging are intricate and not yet fully comprehended. Similar to other complex phenotypes, CKD may arise from a combination of inherited genetic risk variants and environmental exposures.

Our study suggested that individuals with CKD exhibit a reduced life expectancy compared to those without this disease, consistent with previous evidence that a lower level of abnormal kidney function is associated with decreased life expectancy and an increased risk of mortality (Neild, 2017; Turin et al., 2012). Moreover, our results indicated that among people with CKD, accelerated biological aging was associated with increased mortality risk and lost years of life expectancy. Similar impacts of biological aging on life expectancy and mortality risk have been observed across various chronic conditions. A cohort study utilizing NHANES data demonstrated a positive association between biological aging, as measured by KDMAge, PhenoAge, telomere length, and klotho concentration, and mortality risk among individuals with diabetes (Chen et al., 2022). Another NHANES study indicated that KDM Biological Age, homeostatic dysregulation, and LM Biological Age were associated with an increased risk of disability and mortality in older adults (Parker et al., 2020). A previous study showed that DNAmGrimAge and DNAmTL, two blood epigenetic biomarkers, were associated with 1‐year mortality in patients with chronic obstructive pulmonary disease (Hernandez Cordero et al., 2021). Furthermore, we also noted that individuals with CKD and accelerated biological aging exhibited significantly reduced life expectancy at age 45 years. Therefore, monitoring biological aging and implementing anti‐aging measurements in both CKD patients and non‐CKD individuals may potentially mitigate the occurrence and progression of CKD and prevent death.

This study underscores the significance of age‐related changes in organ and system functionality within the pathophysiology of CKD, thereby establishing a scientific background for the manifestation of CKD among midlife and elderly individuals. However, the underlying mechanisms regarding biological aging on CKD development remain elusive, encompassing a spectrum of stages ranging from molecular alterations accumulation to physical impairments and the onset of this disease. One possible explanation is that changes in molecular processes associated with the aging of biological systems, such as telomere erosion and DNA methylation, or physiological changes occurring downstream from the molecular origins of aging, such as accelerated vascular aging, could directly impact psychological mechanisms contributing to CKD (Hobson et al., 2023; Matías‐García et al., 2021; Park et al., 2021). A parallel hypothesis suggests declining physical health or chronic disease resulting from age‐related biology may impair psychological well‐being and play a role in the development of CKD (Lo et al., 2023). In our study, further adjusting for the occurrence of coexisting comorbidities such as diabetes, hypertension, and CVD attenuated the associations between biological age and CKD risk, indicating that the emergence of physical health problems may partially mediate the aforementioned relationship.

To the best of our knowledge, this study was the first to prospectively investigate the associations between biological AAs measured by two well‐established algorithms—KDM‐BA and PhenoAge—and the risk of incident CKD as well as exploring the potential impact of biological AAs on the life expectancy of CKD patients. The major strengths of this study include the prospective cohort design, large sample size, and relatively sufficient biochemistry data for the biological age estimation. Nevertheless, several limitations need to be noted. First, due to the observational nature of our study, the prospective evidence we presented did not provide sufficient support for a direct causal relationship between older biological age and the risk of CKD. It also did not exclude the possibility of a parallel causal pathway in the opposite direction. Thus, to further elucidate the causal relationship or reverse causation between kidney disease and biological aging, it is imperative to access data encompassing repeated evaluations of diverse levels of biological aging along with assessments of renal function and CKD. Second, the UK Biobank data may be subject to healthy volunteer bias, potentially limiting its representativeness of the overall UK population. Thus, the sample we analyzed may therefore have a younger biological age and exhibit lower levels of CKD than the general population (Fry et al., 2017). However, we anticipate that these selection biases will attenuate the association estimates toward null, and our findings are therefore expected to be more conservative. Third, given the potential decline in kidney function with healthy aging and the inclusion of chronological age within the eGFR equation, increasing evidence suggests that the current definition of CKD should be age‐adjusted (Levey et al., 2020; Liu et al., 2021). Accordingly, the current method to define CKD regardless of patient age could result in an overestimation of the CKD burden in an elderly population, thereby inducing potential ascertainment bias. Last, participants in this study were mostly middle‐aged or older individuals of European white descent, which limits the generalization of our findings to other age and ethnic groups.

In summary, this large‐scale prospective cohort study found that biological aging acceleration was associated with an increased risk of CKD, especially among people with high genetic risk for abnormal renal function. Moreover, biological aging acceleration was associated with lower life expectancy among patients with CKD. Under the circumstances of population aging, it is imperative to formulate policies targeting decelerating biological aging starting from midlife to alleviate the burden of CKD and improve life expectancy. Future clinical trials are warranted to investigate potential interventions aimed at attenuating the process of biological aging and evaluating their impact on CKD.

## AUTHOR CONTRIBUTIONS

GZ, QC, Y‐XZ, YX, and Y‐HZ conceived the study. GZ, YX, and Y‐HZ contributed to the design. GZ, QC, and Y‐XZ analyzed the data and drafted the manuscript. All authors interpreted the data, revised the manuscript, and approved the final version. GZ, QC, and Y‐XZ contributed equally to this work.

## FUNDING INFORMATION

This study was supported by the National Key R&D Program (grant number 2023YFC3604605 to Yuhong Zhao), the Young Elite Scientists Sponsorship Program by China Association for Science and Technology (grant number YESS20200151 to Yang Xia), the 345 Talent Project of Shengjing Hospital of China Medical University (M0294 to Yang Xia), and the Scientific Research Project of Liaoning Province Education Department (grant number LJKMZ20221149 to Yang Xia), the LiaoNing Revitalization Talents Program (grant number XLYC2203168 to Yang Xia). The funders had no role in the design and conduct of the study; collection, management, analysis, and interpretation of the data; preparation, review, or approval of the manuscript; and decision to submit the manuscript for publication.

## CONFLICT OF INTEREST STATEMENT

None declared.

## Supporting information


Appendix S1.

## Data Availability

The datasets generated and analyzed during the current study from the UK Biobank Study have been conducted using the UK Biobank Resource under Application Number 63454.
