# Supplementary material for: Associations of clinical parameter‐based accelerated aging, genetic predisposition with risk of chronic kidney disease and associated life expectancy: A prospective cohort study
Source: Aging Cell. 2024 Dec 24;24(4):e14453. doi: 10.1111/acel.14453 (PMC11984662; doi:10.1111/acel.14453)
Supplement: Supplementary file 1 — Appendix S1. [file ACEL-24-e14453-s001.docx]

**Associations of clinical parameter-based accelerated aging, genetic predisposition with risk of chronic kidney disease and associated life expectancy: A prospective cohort study**

Gang Zheng ^1, 2#^, Qing Chang ^1, 2^^#^, Yixiao Zhang ^3#^, Yashu Liu ^4^, Chao Ji ^1, 2^, Honghao Yang ^1, 2^, Liangkai Chen ^5^, Yang Xia ^1, 2*^, Yuhong Zhao ^1, 2*^

**Supplementary Figure S1.** Flowchart for the selection of the study population from UK Biobank study.

**Supplementary Figure S2.** Cumulative incidence function of incident CKD according to quartile groups of KDM-BA acceleration (A) and PhenoAge acceleration (B).

**Supplementary Figure S3.** Associations (HRs and 95% CIs) of KDM-BA acceleration and PhenoAge acceleration with incident CKD by polygenic score for eGFR.

**Supplementary Figure S4.** Subgroup analyses for the associations (HRs and 95% CIs) of KDM-BA acceleration and PhenoAge acceleration with incident CKD in 281,363 participants.

**Supplementary Table S1.** SNPs used for creating the eGFR-PGS in the UK Biobank Study.

**Supplementary Table S2.** Correlation matrix of chronological ages, biological ages, and age accelerations (Pearson correlation).

**Supplementary Table S3.** Associations (HRs and 95% CIs) between the polygenic score of eGFR and incident CKD in 281,363 participants.

**Supplementary Table S4.** Estimates years (95% CIs) of life expectancy and life lost according to KDM-BA acceleration and PhenoAge acceleration among participants with different CKD status at age 45 years.

**Supplementary Table S5.** Associations (HRs and 95% CIs) of KDM-BA acceleration and PhenoAge acceleration with incident CKD restricting to participants with ≥ two years of follow-up (n = 280,206).

**Supplementary Table S6.** Associations (HRs and 95% CIs) of KDM-BA acceleration and PhenoAge acceleration with incident CKD using the sub-distribution competing risk model in 281,363 participants.

**Supplementary Table S7.** Associations (HRs and 95% CIs) of KDM-BA acceleration and PhenoAge acceleration with incident CKD with further adjustments of cardiometabolic biomarkers or eGFR.

**Supplementary Table S8.** Associations (HRs and 95% CIs) of KDM-BA acceleration and PhenoAge acceleration with incident CKD excluding participants with pre-existing cardiovascular disease and diabetes (n = 257,228).

**Supplementary Method**

***Calculation of the GRS for eGFR***

The construction of a polygenic score (PGS) for estimated glomerular filtration rate (eGFR) utilized a total of 263 single nucleotide polymorphisms (SNPs) that have been reported to be significantly associated with eGFR levels (Zhang et al., 2022). Each SNP was assigned a numerical code of 0, 1, or 2 based on the number of risk alleles present. The effect size, represented by the regression coefficient, for each SNP, was obtained from data derived from a genome-wide association study (Zhang et al., 2022). The weighted PGS for eGFR was calculated as the sum of the products between the number of risk alleles and their corresponding regression coefficients: (β1 × SNP1 + β2 × SNP2 + ...+β263 × SNP263), multiplied by (263 divided by the sum of all β coefficients). Previous research studies have extensively employed and validated this calculation method (Lewis & Vassos, 2020). A higher PGS for eGFR indicates an increased genetic predisposition towards elevated eGFR levels, suggesting a lower genetic susceptibility to abnormal renal function.

***Data collection and measurements***

The detailed study protocols have been described in a previous study(Collins, 2012). Sociodemographic data, lifestyle information, and medical history were collected at recruitment. Based on self-reported data, ethnicity was categorized as ‘White’, ‘Black’, and ‘Multiethnic or other’. The Townsend deprivation index was used to measure socioeconomic deprivation, based on the participants' residential postcode, incorporating the information on employment status, ownership of a car and home, and educational attainment was self-reported as follows: college or university degree, A/AS levels or equivalent, O levels/GCSEs or equivalent, and none of those above. The smoking status was categorized as "never," "former," and "current," while the alcohol consumption status was classified as daily or almost daily, 1–4 times a week, 1–3 times a month, or never or only on special occasions. Cardiovascular diseases, diabetes, hypertension, and cancer were defined as self-reported physician-diagnosed cases. Standardized techniques were employed to gather physical measurements and biological specimens and the procedures have previously been described and validated(Collins, 2012). Serum creatinine was measured by the isotope dilution mass spectrometry-traceable method, and the CKD-EPI equation (Chronic Kidney Disease Epidemiology Collaboration) was used to calculate eGFR(Levey et al., 2009). Urine creatinine was measured by the enzymatic method, and urine albumin was measured by the immune-turbidimetric method. Detailed methods for other laboratory measurements were described in the previous study protocol (<http://biobank.ctsu.ox.ac.uk/crystal/label.cgi?id=17518>). Additional information on the measurements is available on the UK Biobank website (<http://www.ukbiobank.ac.uk>).

***Outcome ascertainment***

The incident CKD cases were determined by utilizing International Classification of Diseases, 10th Revision (ICD-10) codes (E10.2, E11.2, I12.x, I13.x, N03.x, N11.x, N18.x, T86.1, Z49.x, Z94.0 and Z99.2) present in primary care data, hospital inpatient data, and death register records as well as Office of Population Censuses and Surveys Classification of Interventions and Procedures-version 4 (OPCS-4) codes ((L74.1–74.6, L74.8–74.9, M01.2–01.9, M02.3, M08.4, M17.2, M17.4,  M17.8–17.9, X40.1–40.9, X41.1–41.2, X41.8-41.9, X42.1, and X42.8-42.9) from hospital inpatient data. The hospital's inpatient records were obtained by establishing connections with the Hospital Episode Statistics for England, Scottish Morbidity Records for Scotland, and the Patient Episode Database for Wales. The identification of CKD-related deaths was accomplished by linking the data to the death registry. Follow-up time was calculated starting from the baseline date until the occurrence of CKD diagnosis, censoring date, or death (up to May 2021), whichever came first. Prevalent CKD was determined by the presence of eGFR <60 mL/min per 1·73m2 or UACR >30 mg/g at recruitment, identification of prevalent cases using the above ICD-10/OPCSR codes, or self-reported CKD cases.

**Reference**

Collins, R. (2012). What makes UK Biobank special? *Lancet*, *379*(9822), 1173-1174. <https://doi.org/10.1016/s0140-6736(12)60404-8>

Levey, A. S., Stevens, L. A., Schmid, C. H., Zhang, Y. L., Castro, A. F., 3rd, Feldman, H. I., Kusek, J. W., Eggers, P., Van Lente, F., Greene, T., & Coresh, J. (2009). A new equation to estimate glomerular filtration rate. *Ann Intern Med*, *150*(9), 604-612. <https://doi.org/10.7326/0003-4819-150-9-200905050-00006>

Lewis, C. M., & Vassos, E. (2020). Polygenic risk scores: from research tools to clinical instruments. *Genome Med*, *12*(1), 44. <https://doi.org/10.1186/s13073-020-00742-5>

Zhang, H., Wang, B., Chen, C., Sun, Y., Chen, J., Tan, X., Xia, F., Zhang, J., Lu, Y., & Wang, N. (2022). Sleep Patterns, Genetic Susceptibility, and Incident Chronic Kidney Disease: A Prospective Study of 370 671 Participants. *Front Neurosci*, *16*, 725478. <https://doi.org/10.3389/fnins.2022.725478>


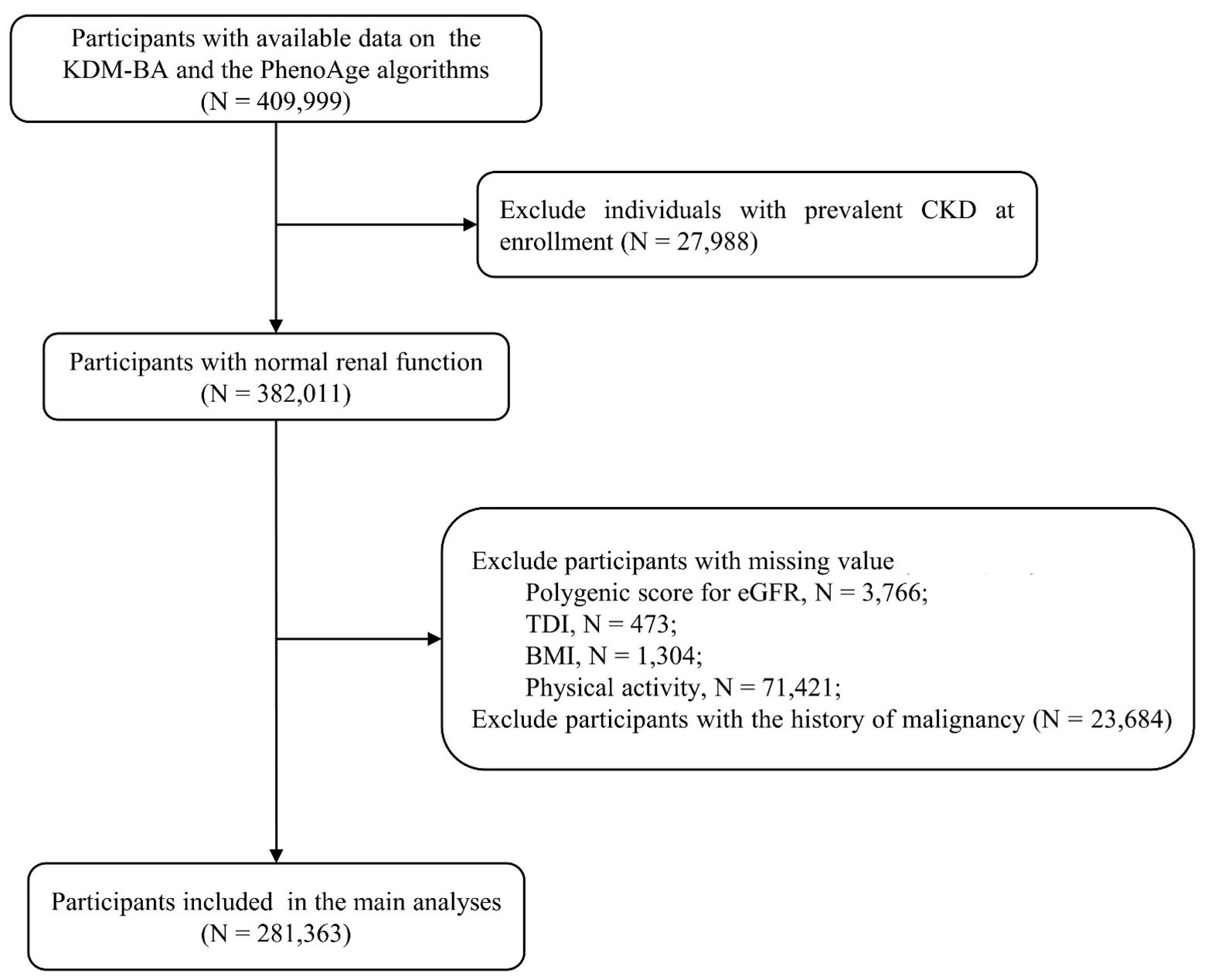


**Supplementary Figure S1. Flowchart for the selection of the study population from UK Biobank study.** BMI, body mass index; eGFR, estimated glomerular filtration rate; TDI, Townsend Deprivation Index.


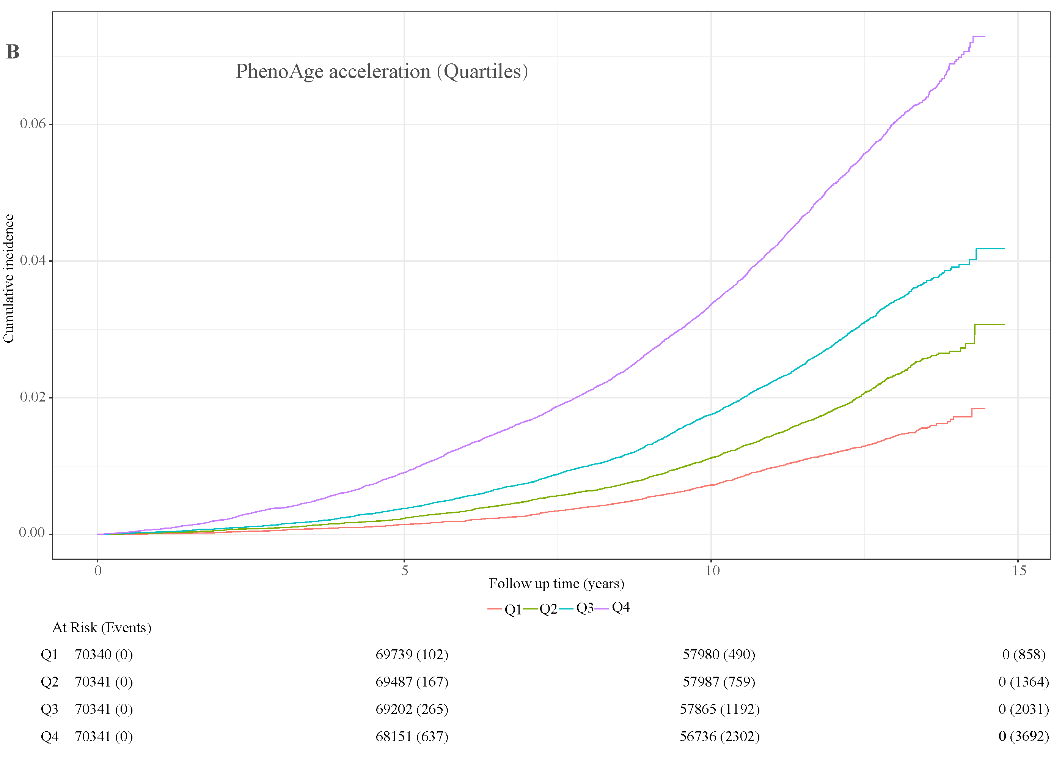

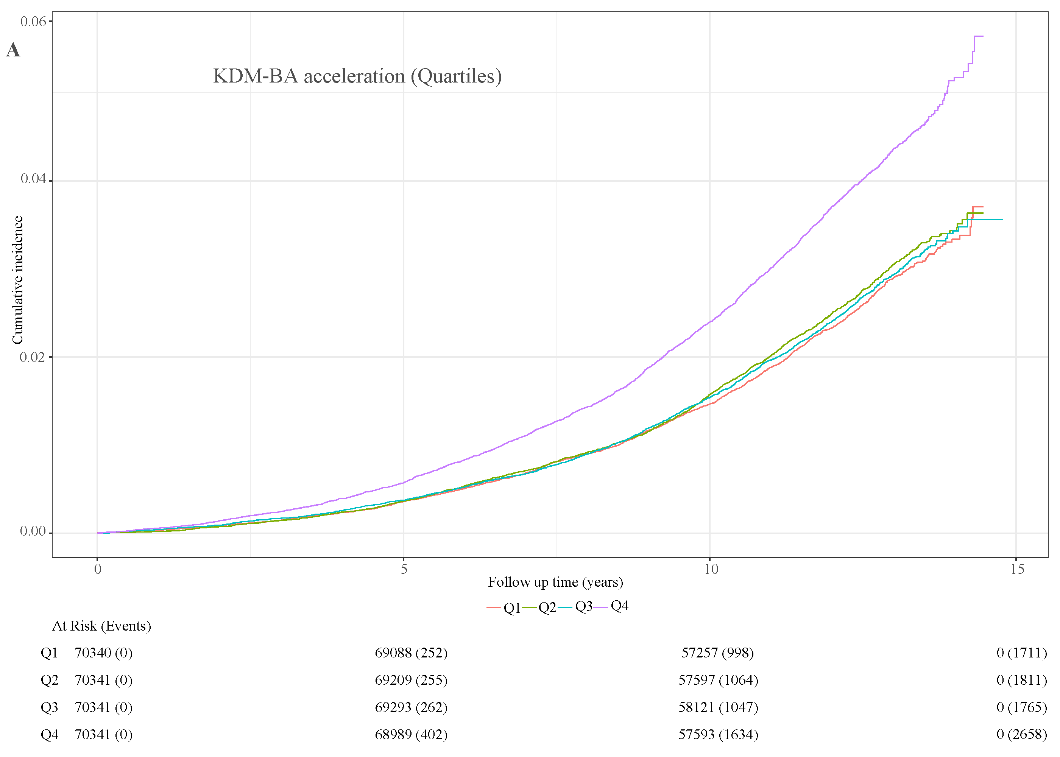
**Supplementary** **Figure S2. Cumulative incidence function of incident CKD according to quartile groups of KDM-BA acceleration (A) and PhenoAge acceleration (B).**

**Supplementary Figure S3. Associations (HRs and 95% CIs) of** **KDM-BA acceleration and PhenoAge acceleration with incident CKD by polygenic score for eGFR*.** CIs, confidence intervals; CKD, chronic kidney disease; eGFR, estimated glomerular filtration rate; HRs, hazard ratios; Q, quintile; ref, reference group; SD, standard deviation. HRs were calculated from cause-specific competing risk models adjusted for age (years, continuous), sex (male, female), race (White, Asian, Black, Mixed), BMI (kg/m^2^, continuous), Townsend deprivation index (continuous), smoking status (never, previous, current), alcohol consumption (daily or almost daily, 1–4 times a week, 1–3 times a month, never or special occasions only), education (college or university degree, high school, below), physical activity (MET-min/week, continuous), and history of hypertension, cardiovascular disease, and diabetes (yes, no).

*****A high polygenic score for eGFR indicates a lower genetic risk of abnormal renal function, with a low polygenic score indicating a higher genetic risk.

**Supplementary Figure S4. Subgroup analyses for the associations (HRs and 95% CIs) of KDM-BA acceleration and PhenoAge acceleration with incident CKD in** **281,363 participants.** BMI, body mass index; CIs, confidence intervals; HRs, hazard ratios; SD, standard deviation. HRs were calculated from cause-specific competing risk models adjusted for age (years, continuous), sex (male, female), race (White, Asian, Black, Mixed), BMI (kg/m^2^, continuous), Townsend deprivation index (continuous), smoking status (never, previous, current), alcohol consumption (daily or almost daily, 1–4 times a week, 1–3 times a month, never or special occasions only), education (college or university degree, high school, below), physical activity (MET-min/week, continuous), and history of hypertension, cardiovascular disease, and diabetes (yes, no).

**Supplementary Table S1. SNPs used for creating the eGFR-PGS in the UK Biobank Study.**

| SNP | Effect Allele | Chr | Effect | Locus |
| --- | --- | --- | --- | --- |
| rs61830291 | a | 1 | -0.0036 | LINC01352 |
| rs2490391 | a | 1 | -0.0024 | SDCCAG8 |
| rs12061708 | a | 1 | -0.0026 | KLHDC7A |
| rs2749153 | a | 1 | -0.0033 | ZNF436-AS1 |
| rs688540 | a | 1 | -0.0030 | FOXD2 |
| rs3845534 | a | 1 | -0.0019 | LOC100422212 |
| rs1011731 | a | 1 | -0.0019 | DNM3 |
| rs78444298 | a | 1 | -0.0105 | EDEM3 |
| rs78329830 | a | 1 | -0.0054 | PLA2G4A |
| rs1887252 | c | 1 | -0.0019 | LINC01362 |
| rs7543734 | c | 1 | 0.0031 | BCAR3 |
| rs74748843 | t | 1 | -0.0048 | CASZ1 |
| rs659437 | t | 1 | -0.0027 | AKR1A1 |
| rs10159261 | t | 1 | -0.0034 | AGMAT |
| rs267738 | t | 1 | -0.0048 | CERS2 |
| rs17413465 | a | 1 | 0.0025 | MIR4422HG |
| rs11211257 | a | 1 | 0.0027 | PIK3R3 |
| rs1757915 | a | 1 | 0.0021 | LINC01755 |
| rs11166440 | a | 1 | 0.0020 | CDC14A |
| rs10857788 | a | 1 | 0.0030 | SYPL2 |
| rs4971100 | a | 1 | 0.0020 | TRIM46 |
| rs3850625 | a | 1 | 0.0046 | CACNA1S |
| rs2808454 | a | 1 | 0.0019 | PFKFB2 |
| rs12736457 | c | 1 | 0.0054 | PPM1J |
| rs75625374 | c | 1 | 0.0045 | CD34 |
| rs7536433 | t | 1 | 0.0021 | AK5 |
| rs679843 | t | 1 | 0.0021 | MGC27382 |
| rs3118119 | t | 1 | 0.0030 | LOC105371433 |
| rs4656220 | t | 1 | 0.0020 | PRRX1 |
| rs3795503 | t | 1 | 0.0020 | KIAA1614 |
| rs7535253 | t | 1 | 0.0021 | PTPN14 |
| rs2577134 | t | 1 | 0.0020 | RNU5F-1 |
| rs417237 | t | 1 | 0.0018 | OBSCN |
| rs1047891 | a | 2 | -0.0065 | CPS1 |
| rs17050272 | a | 2 | -0.0022 | LINC01101 |
| rs2971880 | a | 2 | -0.0024 | SPTBN1 |
| rs60980181 | a | 2 | -0.0027 | CALCRL |
| rs4664475 | t | 2 | -0.0020 | NEB |
| rs35472707 | t | 2 | -0.0073 | LRP2 |
| rs2301343 | t | 2 | -0.0023 | SLC8A1 |
| rs35284526 | a | 2 | 0.0029 | NFE2L2 |
| rs4491726 | a | 2 | 0.0032 | RDH14 |
| rs6546869 | a | 2 | 0.0059 | ALMS1P1 |
| rs11694902 | a | 2 | 0.0041 | TFCP2L1 |
| rs7425436 | a | 2 | 0.0024 | ORC4 |
| rs35669853 | a | 2 | 0.0024 | MIR5702 |
| rs10197255 | a | 2 | 0.0018 | LINC01812 |
| rs10865189 | c | 2 | 0.0024 | ZFP36L2 |
| rs187355703 | c | 2 | 0.0100 | HOXD8 |
| rs780093 | t | 2 | 0.0044 | GCKR |
| rs11123169 | t | 2 | 0.0025 | PSD4 |
| rs1548945 | t | 2 | 0.0036 | TNP1 |
| rs1050816 | t | 2 | 0.0026 | SPEG |
| rs13003198 | t | 2 | 0.0018 | SAG |
| rs807624 | t | 2 | 0.0032 | DDX1 |
| rs4666821 | t | 2 | 0.0020 | PDE1A |
| rs3791221 | a | 2 | 0.0022 | SH3YL1 |
| rs6779998 | a | 3 | -0.0017 | TGFBR2 |
| rs4625 | a | 3 | -0.0023 | DAG1 |
| rs9828976 | c | 3 | -0.0024 | SLC35G2 |
| rs56065557 | c | 3 | -0.0029 | SENP2 |
| rs6778731 | t | 3 | -0.0017 | WNT7A |
| rs3774726 | t | 3 | -0.0021 | ATXN7 |
| rs2289746 | t | 3 | -0.0019 | CBLB |
| rs35320690 | t | 3 | -0.0025 | MSL2 |
| rs11919484 | t | 3 | -0.0026 | KNG1 |
| rs2581820 | a | 3 | 0.0021 | SFMBT1 |
| rs9868185 | a | 3 | 0.0026 | SLC15A2 |
| rs1397764 | a | 3 | 0.0043 | TFDP2 |
| rs9823161 | a | 3 | 0.0022 | LINC02028 |
| rs11914389 | t | 3 | 0.0030 | ACVR2B |
| rs7651407 | t | 3 | 0.0025 | PLXNB1 |
| rs10934754 | t | 3 | 0.0020 | ALDH1L1-AS2 |
| rs7624084 | t | 3 | 0.0017 | ZBTB38 |
| rs76272256 | t | 3 | 0.0024 | MECOM |
| rs795009 | t | 3 | 0.0020 | SYN2 |
| rs3775932 | a | 4 | -0.0018 | WDR1 |
| rs16874073 | t | 4 | -0.0045 | PPARGC1A |
| rs4864890 | t | 4 | -0.0023 | DCUN1D4 |
| rs12509595 | t | 4 | -0.0035 | FGF5 |
| rs75501914 | a | 4 | 0.0039 | HGFAC |
| rs71606723 | a | 4 | 0.0025 | UGT8 |
| rs223471 | c | 4 | 0.0028 | LOC102723704 |
| rs55929207 | c | 4 | 0.0019 | ETNPPL |
| rs28817415 | t | 4 | -0.0073 | SHROOM3 |
| rs12163971 | a | 5 | -0.0029 | AFF4 |
| rs13159523 | a | 5 | -0.0024 | TPPP |
| rs13157326 | a | 5 | -0.0027 | RAI14 |
| rs2010352 | a | 5 | -0.0018 | AK6 |
| rs1362800 | t | 5 | -0.0049 | DAB2 |
| rs3812036 | t | 5 | -0.0065 | SLC34A1 |
| rs72759880 | t | 5 | -0.0056 | PIK3R1 |
| rs3797537 | a | 5 | 0.0019 | DMGDH |
| rs12520984 | c | 5 | 0.0019 | FST |
| rs12777 | c | 5 | 0.0050 | SLC22A4 |
| rs11746506 | t | 5 | 0.0017 | MRPS30 |
| rs11743174 | t | 5 | 0.0019 | ABLIM3 |
| rs495237 | t | 5 | 0.0027 | LINC00603 |
| rs79760705 | t | 5 | 0.0056 | ARL15 |
| rs881858 | a | 6 | -0.0054 | LINC01512 |
| rs72912510 | a | 6 | -0.0024 | RRAGD |
| rs9375818 | a | 6 | -0.0031 | ARG1 |
| rs3822939 | a | 6 | -0.0025 | EYA4 |
| rs12207180 | a | 6 | -0.0085 | SLC22A2 |
| rs12212034 | t | 6 | -0.0018 | PKHD1 |
| rs6458868 | t | 6 | -0.0020 | GSTA2 |
| rs3925003 | t | 6 | -0.0018 | HMGCLL1 |
| rs13200335 | a | 6 | 0.0024 | TFEB |
| rs11755724 | a | 6 | 0.0027 | RREB1 |
| rs1857859 | a | 6 | 0.0019 | SIM1 |
| rs1268168 | a | 6 | 0.0024 | FOXO3 |
| rs9397738 | a | 6 | 0.0027 | SCAF8 |
| rs77915916 | a | 6 | 0.0046 | CRIP3 |
| rs7740107 | a | 6 | 0.0027 | L3MBTL3 |
| rs3765502 | t | 6 | 0.0024 | DCDC2 |
| rs144100226 | t | 6 | 0.0059 | HMGA1 |
| rs720989 | t | 6 | 0.0021 | SUPT3H |
| rs35154268 | a | 7 | -0.0022 | SND1 |
| rs6968554 | a | 7 | -0.0019 | AHR |
| rs62491533 | t | 7 | -0.0027 | UBE2H |
| rs10254101 | t | 7 | -0.0068 | PRKAG2 |
| rs62435145 | t | 7 | -0.0060 | UNCX |
| rs3750081 | t | 7 | -0.0022 | KBTBD2 |
| rs801193 | t | 7 | -0.0020 | GS1-124K5.11 |
| rs6973656 | a | 7 | 0.0035 | TMEM60 |
| rs700753 | c | 7 | 0.0031 | LOC730338 |
| rs55773927 | t | 7 | 0.0019 | VKORC1L1 |
| rs41301394 | t | 7 | 0.0023 | POR |
| rs3757387 | t | 7 | 0.0030 | IRF5 |
| rs12671694 | t | 7 | 0.0025 | SHH |
| rs868822 | t | 7 | 0.0029 | LINC01006 |
| rs11783418 | a | 8 | -0.0020 | XKR6 |
| rs10102889 | c | 8 | -0.0036 | NRG1 |
| rs2976178 | c | 8 | -0.0025 | WWP1 |
| rs2980423 | t | 8 | -0.0023 | PRAG1 |
| rs35353426 | t | 8 | -0.0026 | LOC157273 |
| rs10098664 | t | 8 | -0.0021 | BLK |
| rs34861762 | t | 8 | -0.0043 | STC1 |
| rs1533059 | a | 8 | 0.0025 | MFHAS1 |
| rs7832708 | t | 8 | 0.0022 | MSRA |
| rs2954017 | t | 8 | 0.0024 | TRIB1 |
| rs12377027 | a | 9 | -0.0026 | MLLT3 |
| rs13287724 | a | 9 | -0.0030 | B4GALT1-AS1 |
| rs1321917 | c | 9 | -0.0023 | ASTN2 |
| rs544169 | a | 9 | 0.0022 | UBAP2 |
| rs2039424 | a | 9 | 0.0044 | PIP5K1B |
| rs7024579 | t | 9 | 0.0023 | QSOX2 |
| rs2068888 | a | 10 | -0.0024 | CYP26A1 |
| rs4918943 | a | 10 | -0.0022 | SORBS1 |
| rs12240572 | a | 10 | -0.0032 | DNAJC9-AS1 |
| rs7095954 | a | 10 | -0.0018 | TSPAN14 |
| rs816850 | c | 10 | -0.0020 | KCNMA1 |
| rs1536225 | t | 10 | -0.0021 | PDCD11 |
| rs7072591 | a | 10 | 0.0019 | PARD3-AS1 |
| rs10821905 | a | 10 | 0.0037 | A1CF |
| rs1055256 | a | 10 | 0.0025 | EEF1AKMT2 |
| rs80282103 | a | 10 | 0.0078 | LARP4B |
| rs6481598 | c | 10 | 0.0024 | SVIL |
| rs8474 | c | 10 | 0.0020 | PARG |
| rs7475348 | t | 10 | 0.0031 | MYPN |
| rs9420446 | t | 10 | 0.0023 | FAM35A |
| rs10821944 | t | 10 | 0.0020 | ARID5B |
| rs284859 | t | 10 | 0.0026 | WBP1L |
| rs1541937 | a | 11 | -0.0029 | OR52H1 |
| rs1783827 | a | 11 | -0.0020 | MIR130A |
| rs3892895 | a | 11 | -0.0023 | TPCN2 |
| rs963837 | t | 11 | -0.0057 | DCDC1 |
| rs6484504 | t | 11 | -0.0026 | DNAJC24 |
| rs2727040 | t | 11 | -0.0026 | TRIM49B |
| rs948493 | t | 11 | -0.0033 | MIR1234 |
| rs11237450 | a | 11 | 0.0032 | GAB2 |
| rs63934 | a | 11 | 0.0041 | KCNQ1 |
| rs6589750 | a | 11 | 0.0020 | USP2-AS1 |
| rs11564722 | t | 11 | 0.0033 | INS-IGF2 |
| rs61897431 | t | 11 | 0.0029 | SLC39A13 |
| rs7127946 | t | 11 | 0.0023 | OR4B1 |
| rs1813937 | t | 11 | 0.0022 | LOC646813 |
| rs10790452 | t | 11 | 0.0020 | SORL1 |
| rs10846157 | a | 12 | -0.0034 | RERG |
| rs11062167 | a | 12 | -0.0039 | SLC6A13 |
| rs632887 | a | 12 | 0.0032 | TSPAN9 |
| rs117113238 | a | 12 | 0.0039 | BCL2L14 |
| rs2634675 | a | 12 | 0.0025 | ZNF641 |
| rs1275609 | a | 12 | 0.0024 | PHLDA1 |
| rs4238020 | t | 12 | 0.0029 | C12orf4 |
| rs12313306 | t | 12 | 0.0029 | R3HDM2 |
| rs41284816 | t | 13 | -0.0078 | DLEU2 |
| rs500830 | t | 13 | 0.0029 | DACH1 |
| rs61993680 | a | 14 | -0.0019 | SLC25A29 |
| rs72683923 | t | 14 | -0.0074 | L2HGDH |
| rs6574652 | t | 14 | -0.0017 | STON2 |
| rs17184313 | t | 14 | -0.0029 | RIN3 |
| rs1028455 | a | 14 | 0.0020 | SPATA7 |
| rs690428 | a | 15 | -0.0039 | WDR72 |
| rs1994887 | a | 15 | -0.0020 | CGNL1 |
| rs351237 | a | 15 | -0.0018 | STRA6 |
| rs4886696 | a | 15 | -0.0032 | SIN3A |
| rs6492982 | t | 15 | -0.0033 | INO80 |
| rs11071738 | t | 15 | -0.0025 | APH1B |
| rs11071939 | t | 15 | -0.0039 | SMAD3 |
| rs1145077 | t | 15 | -0.0085 | GATM |
| rs59646751 | t | 15 | -0.0023 | IGF1R |
| rs4886755 | a | 15 | 0.0041 | NRG4 |
| rs17507300 | a | 15 | 0.0024 | BTBD1 |
| rs7169629 | c | 15 | 0.0018 | WDR73 |
| rs12913015 | t | 15 | 0.0027 | C15orf54 |
| rs956006 | t | 15 | 0.0019 | MGC15885 |
| rs2472297 | t | 15 | 0.0039 | CYP1A1 |
| rs166906 | t | 15 | 0.0033 | SCAPER |
| rs9932625 | a | 16 | -0.0030 | LINC01571 |
| rs28581385 | a | 16 | -0.0028 | LINC01229 |
| rs154656 | a | 16 | -0.0030 | CHMP1A |
| rs1635404 | t | 16 | -0.0025 | TRAP1 |
| rs193538 | t | 16 | -0.0020 | ABCC1 |
| rs7185391 | t | 16 | -0.0027 | SLC7A6 |
| rs62053077 | t | 16 | -0.0021 | MARVELD3 |
| rs7203398 | a | 16 | 0.0025 | CHD9 |
| rs77924615 | a | 16 | 0.0098 | PDILT |
| rs62050038 | a | 16 | 0.0028 | WWP2 |
| rs438339 | t | 16 | 0.0035 | RPL3L |
| rs1858800 | t | 16 | 0.0020 | ZFHX3 |
| rs883541 | a | 17 | -0.0022 | PRKAR1A |
| rs2411192 | a | 17 | -0.0024 | MYO19 |
| rs8866 | c | 17 | -0.0018 | PITPNC1 |
| rs2349648 | t | 17 | -0.0017 | MPRIP |
| rs4794813 | a | 17 | 0.0055 | CDK12 |
| rs35662455 | c | 17 | 0.0030 | TEX14 |
| rs9903801 | c | 17 | 0.0047 | BCAS3 |
| rs9891340 | t | 17 | 0.0024 | SMCR2 |
| rs2440165 | t | 17 | 0.0040 | SLC47A1 |
| rs9895661 | t | 17 | 0.0069 | BCAS3 |
| rs28735420 | t | 17 | 0.0039 | MAP2K4 |
| rs227731 | t | 17 | 0.0018 | C17orf67 |
| rs16942751 | a | 18 | -0.0029 | AQP4 |
| rs1719934 | a | 18 | 0.0026 | EPB41L3 |
| rs8096658 | c | 18 | 0.0050 | NFATC1 |
| rs4940525 | t | 18 | 0.0025 | LINC01544 |
| rs34647824 | a | 19 | -0.0021 | RRAS |
| rs78241494 | t | 19 | -0.0030 | ZNF585A |
| rs281380 | t | 19 | -0.0021 | MAMSTR |
| rs2974751 | a | 19 | 0.0018 | CALR |
| rs8101667 | t | 19 | 0.0044 | CEP89 |
| rs7251730 | t | 19 | 0.0024 | ZNF260 |
| rs113445505 | t | 19 | 0.0037 | ZNF781 |
| rs6087579 | a | 20 | -0.0028 | ITCH |
| rs4408777 | a | 20 | -0.0021 | RGS19 |
| rs2235826 | a | 20 | -0.0030 | PCK1 |
| rs1041606 | t | 20 | -0.0021 | MACROD2 |
| rs17216707 | t | 20 | -0.0051 | CYP24A1 |
| rs1509117 | a | 20 | 0.0024 | PLCB1 |
| rs72629024 | c | 20 | 0.0035 | PPDPF |
| rs62187537 | t | 20 | 0.0039 | FKBP1ASDCBP2 |
| rs1407040 | t | 20 | 0.0018 | GNAS |
| rs35636653 | t | 20 | 0.0022 | OSBPL2 |
| rs2273684 | t | 20 | 0.0032 | GSS |
| rs2823139 | a | 21 | -0.0026 | NRIP1 |
| rs2834317 | a | 21 | -0.0035 | LOC101928126 |
| rs2244237 | t | 21 | 0.0027 | CLDN14 |
| rs80576 | a | 22 | -0.0028 | APOL3 |
| rs4820324 | c | 22 | -0.0023 | MAFF |
| rs131263 | t | 22 | 0.0024 | ZMAT5 |
| rs112880707 | t | 22 | 0.0052 | MKL1 |
| rs738527 | t | 22 | 0.0032 | A4GALT |

*Abbreviations:* Chr, chromosome; eGFR, estimated glomerular filtration rate; PGS, polygenic score; SNP, single nucleotide polymorphism.

**Supplementary Table S2. Correlation matrix of chronological ages, biological ages, and age accelerations (Pearson correlation).**

| **Characteristics** | **Mean** | **standard deviation** | **Age** | **KDM-BA** | **KDM-BA acceleration** | **PhenoAge** | **PhenoAge acceleration** |
| --- | --- | --- | --- | --- | --- | --- | --- |
| **Age** | 55.8 | 8.1 | 1 | 0.40 (*P* <0.0001) | -0.06 (*P* <0.0001) | 0.85 (*P* <0.0001) | 0.04 (*P* <0.0001) |
| **KDM-BA** | 40.2 | 17.7 | - | 1 | 0.89 (*P* <0.0001) | 0.46 (*P* <0.0001) | 0.23 (*P* <0.0001) |
| **KDM-BA acceleration** | -15.6 | 16.3 | - | - | 1 | 0.07 (*P* <0.0001) | 0.23 (*P* <0.0001) |
| **PhenoAge** | 44.7 | 9.8 | - | - | - | 1 | 0.57 (*P* <0.0001) |
| **PhenoAge acceleration** | -11.2 | 5.2 | - | - | - | - | 1 |

**Supplementary Table S3. Associations (HRs and 95% CIs) between the polygenic score of eGFR and incident CKD** **in 281,363 participants*.**

| **Polygenic score of eGFR** | **Cases/participants** | **Model 1** | **Model 2** | **Model 3** |
| --- | --- | --- | --- | --- |
|  |  | **HR (95% CI)** | **HR (95% CI)** | **HR (95% CI)** |
| **Per** **standard deviation increase** | **-** | 0.73 (0.71, 0.76) | 0.71 (0.68, 0.75) | 0.72 (0.69, 0.74) |
| **Low** **genetically predicted eGFR ^a^** | 4,596/140,679 | 1.00 (Ref) | 1.00 (Ref) | 1.00 (Ref) |
| **High genetically predicted eGFR ^b^** | 3,349/140,684 | 0.73 (0.69, 0.76) | 0.72 (0.70, 0.75) | 0.71 (0.68, 0.74) |

*****HR and 95% CI were calculated using the cause-specific competing risk model.

Model 1 was not adjusted.

Model 2: adjusted for age (years, continuous), sex (male, female), race (White, Asian, Black, Mixed), BMI (kg/m^2^, continuous), Townsend deprivation index (continuous), smoking status (never, previous, current), alcohol consumption (daily or almost daily, 1–4 times a week, 1–3 times a month, never or special occasions only), education (college or university degree, high school, below), physical activity (MET-min/week, continuous), and first 10 genetic principal components, and genotyping batch.

Model 3: Model 2 + history of hypertension, cardiovascular disease, diabetes (yes, no).

**^a^** Low genetically predicted eGFR (below the median of the polygenic score for eGFR).

**^b^** High genetically predicted eGFR (above the median of the polygenic score for eGFR).

*Abbreviations:* CIs, confidence intervals; CKD, chronic kidney disease; eGFR, estimated glomerular filtration rate; HRs, hazard ratios.

**Supplementary Table S4. Estimates years (95% CIs) of** **life expectancy and life lost according to KDM-BA acceleration and PhenoAge acceleration among participants with different CKD status at age 45 years.**

| **Characteristics** | **Participants with CKD** | | **Participants without CKD** | |
| --- | --- | --- | --- | --- |
|  | **Life expectancy** | **Years of life lost** | **Life expectancy** | **Years of life lost** |
| **All participants** | 32.5 (32.0, 33.0) | 5.8 (5.3, 6.2) | 38.3 (37.8, 38.8) | Ref |
| **KDM-BA acceleration** |  |  |  |  |
| Quartile 1 | 27.4 (26.7, 28.1) | Ref | 41.0 (40.4, 41.6) | Ref |
| Quartile 2 | 27.5 (26.8, 28.3) | -0.1 (-0.6, 0.4) | 40.2 (39.6, 40.7) | 0.8 (0.5, 1.1) |
| Quartile 3 | 27.3 (26.6, 28.0) | 0.1 (-0.4, 0.6) | 39.2 (38.6, 39.8) | 1.8 (1.5, 2.2) |
| Quartile 4 | 26.6 (26.0, 27.2) | 0.8 (0.3, 1.4) | 37.3 (36.7, 37.8) | 3.7 (3.3, 4.1) |
| **PhenoAge acceleration** |  |  |  |  |
| Quartile 1 | 27.5 (26.7, 28.3) | Ref | 41.3 (40.7, 41.9) | Ref |
| Quartile 2 | 27.9 (27.1, 28.7) | -0.4 (-1.1, 0.4) | 40.6 (40.0, 41.2) | 0.7 (0.3, 1.0) |
| Quartile 3 | 27.7 (27.0, 28.4) | -0.2 (-0.9, 0.5) | 39.4 (38.8, 40.0) | 1.9 (1.6, 2.3) |
| Quartile 4 | 26.3 (25.8, 26.9) | 1.2 (0.5, 1.9) | 36.1 (35.6, 36.6) | 5.2 (4.7, 5.6) |

**Supplementary Table S5. Associations (HRs and 95% CIs) of KDM-BA acceleration and PhenoAge acceleration with incident CKD restricting to participants with** **≥ two years of follow-up (n = 280,206).**

| **Biological ages** | **Cases (n, %)** | **Model 1** | **Model 2** | **Model 3** |
| --- | --- | --- | --- | --- |
|  |  | **HR (95% CI)** | **HR (95% CI)** | **HR (95% CI)** |
| **KDM-BA acceleration (Quartiles)** | | | | |
| Q1 | 1,653 (2.36%) | 1.00 (Ref) | 1.00 (Ref) | 1.00 (Ref) |
| Q2 | 1,759 (2.51%) | 1.06 (0.99, 1.13) | 1.22 (1.14, 1.31) | 1.21 (1.13, 1.30) |
| Q3 | 1,700 (2.43%) | 1.01 (0.95, 1.08) | 1.36 (1.26, 1.46) | 1.35 (1.25, 1.46) |
| Q4 | 2,561 (3.66%) | 1.54 (1.45, 1.64) | 1.91 (1.77, 2.06) | 1.90 (1.77, 2.05) |
| *P*-trend^a^ |  | <0.0001 | <0.0001 | <0.0001 |
| **Continuous (per SD increment)** |  | 1.19 (1.16, 1.22) | 1.29 (1.26, 1.33) | 1.29 (1.26, 1.33) |
| **PhenoAge acceleration (Quartiles)** | | | | |
| Q1 | 835 (1.19%) | 1.00 (Ref) | 1.00 (Ref) | 1.00 (Ref) |
| Q2 | 1,318 (1.88%) | 1.59 (1.46, 1.74) | 1.36 (1.25, 1.48) | 1.37 (1.25, 1.49) |
| Q3 | 1,965 (2.81%) | 2.40 (2.21, 2.60) | 1.81 (1.67, 1.97) | 1.82 (1.68, 1.98) |
| Q4 | 3,555 (5.07%) | 4.45 (4.13, 4.80) | 2.94 (2.71, 3.18) | 2.75 (2.54, 2.98) |
| *P*-trend^a^ |  | <0.0001 | <0.0001 | <0.0001 |
| **Continuous (per SD increment)** |  | 1.58 (1.56, 1.61) | 1.46 (1.44, 1.49) | 1.41 (1.39, 1.44) |

*****HRs and 95% CIs were calculated with the use of the cause-specific competing risk model.

Model 1 was not adjusted.

Model 2: adjusted for age (years, continuous), sex (male, female), race (White, Asian, Black, Mixed), BMI (kg/m^2^, continuous), Townsend deprivation index (continuous), smoking status (never, previous, current), alcohol consumption (daily or almost daily, 1–4 times a week, 1–3 times a month, never or special occasions only), education (college or university degree, high school, below), and physical activity (MET-min/week, continuous).

Model 3: Model 2 + history of hypertension, cardiovascular disease, diabetes (yes, no).

**^a^** Test for trend based on variables containing the median value for each quartile.

*Abbreviations:* CIs, confidence intervals; CKD, chronic kidney disease; HRs, hazard ratios; SD, standard deviation.

**Supplementary Table S6. Associations (HRs and 95% CIs) of KDM-BA acceleration and PhenoAge acceleration with incident CKD using the sub-distribution competing risk model in 281,363 participants.**

| **Biological ages** | **Cases (n, %)** | **Model 1** | **Model 2** | **Model 3** |
| --- | --- | --- | --- | --- |
|  |  | **HR (95% CI)** | **HR (95% CI)** | **HR (95% CI)** |
| **KDM-BA acceleration (Quartiles)** | | | | |
| Q1 | 1,711 (2.43%) | 1.00 (Ref) | 1.00 (Ref) | 1.00 (Ref) |
| Q2 | 1,811 (2.57%) | 1.06 (0.99, 1.13) | 1.21 (1.13, 1.29) | 1.20 (1.12, 1.28) |
| Q3 | 1,765 (2.51%) | 1.03 (0.96, 1.10) | 1.34 (1.24, 1.44) | 1.33 (1.23, 1.43) |
| Q4 | 2,658 (3.78%) | 1.55 (1.46, 1.65) | 1.86 (1.72, 2.00) | 1.84 (1.70, 1.98) |
| *P*-trend^a^ |  | <0.0001 | <0.0001 | <0.0001 |
| **Continuous (per SD increment)** |  | 1.19 (1.16, 1.22) | 1.28 (1.24, 1.32) | 1.27 (1.24, 1.31) |
| **PhenoAge acceleration (Quartiles)** | | | | |
| Q1 | 858 (1.22%) | 1.00 (Ref) | 1.00 (Ref) | 1.00 (Ref) |
| Q2 | 1,364 (1.94%) | 1.60 (1.47, 1.74) | 1.37 (1.26, 1.49) | 1.38 (1.26, 1.50) |
| Q3 | 2,031 (2.89%) | 2.38 (2.20, 2.58) | 1.82 (1.67, 1.97) | 1.82 (1.68, 1.98) |
| Q4 | 3,692 (5.25%) | 4.36 (4.05, 4.70) | 2.88 (2.67, 3.12) | 2.70 (2.49, 2.92) |
| *P*-trend^a^ |  | <0.0001 | <0.0001 | <0.0001 |
| **Continuous (per SD increment)** |  | 1.56 (1.53, 1.59) | 1.43 (1.41, 1.46) | 1.38 (1.36, 1.41) |

*****HR and 95% CI were calculated with the use of the sub-distribution competing risk model (Fine and Gray’s model).

Model 1 was not adjusted.

Model 2: adjusted for age (years, continuous), sex (male, female), race (White, Asian, Black, Mixed), BMI (kg/m^2^, continuous), Townsend deprivation index (continuous), smoking status (never, previous, current), alcohol consumption (daily or almost daily, 1–4 times a week, 1–3 times a month, never or special occasions only), education (college or university degree, high school, below), and physical activity (MET-min/week, continuous).

Model 3: Model 2 + history of hypertension, cardiovascular disease, diabetes (yes, no).

**^a^** Test for trend based on variables containing the median value for each quartile.

*Abbreviations:* CIs, confidence intervals; CKD, chronic kidney disease; HRs, hazard ratios; SD, standard deviation.

**Supplementary Table S7. Associations (HRs and 95% CIs) of KDM-BA acceleration and PhenoAge acceleration with incident CKD with further adjustments of** **cardiometabolic biomarkers or eGFR.**

| **Model 4 (N = 280,933)** | | **Model 5 (N = 281,363)** | |
| --- | --- | --- | --- |
| **Biological ages** | **HR (95% CI)** | **Biological ages** | **HR (95% CI)** |
| **KDM-BA acceleration (Quartiles)** |  | **KDM-BA acceleration (Quartiles)** |  |
| Q1 | 1.00 (Ref) | Q1 | 1.00 (Ref) |
| Q2 | 1.19 (1.12, 1.28) | Q2 | 1.10 (1.03, 1.18) |
| Q3 | 1.33 (1.23, 1.43) | Q3 | 1.19 (1.10, 1.28) |
| Q4 | 1.88 (1.74, 2.03) | Q4 | 1.49 (1.38, 1.60) |
| *P*-trend^a^ | <0.0001 | *P*-trend^a^ | <0.0001 |
| **Continuous (per SD increment)** | 1.29 (1.26, 1.33) | **Continuous (per SD increment)** | 1.17 (1.14, 1.20) |
| **PhenoAge acceleration (Quartiles)** |  | **PhenoAge acceleration (Quartiles)** |  |
| Q1 | 1.00 (Ref) | Q1 | 1.00 (Ref) |
| Q2 | 1.37 (1.26, 1.50) | Q2 | 1.11 (1.02, 1.21) |
| Q3 | 1.83 (1.68, 1.98) | Q3 | 1.28 (1.18, 1.39) |
| Q4 | 2.76 (2.55, 3.00) | Q4 | 1.66 (1.53, 1.80) |
| *P*-trend^a^ | <0.0001 | *P*-trend^a^ | <0.0001 |
| **Continuous (per SD increment)** | 1.44 (1.42, 1.47) | **Continuous (per SD increment)** | 1.27 (1.24, 1.30) |

*****HRs and 95% CIs were calculated with the use of the cause-specific competing risk model.

Participants with missing value on cardiometabolic biomarkers (triglycerides, LDL-c, hs-CRP) or eGFR was excluded.

Model 4 was adjusted for age (years, continuous), sex (male, female), race (White, Asian, Black, Mixed), BMI (kg/m^2^, continuous), Townsend deprivation index (continuous), smoking status (never, previous, current), alcohol consumption (daily or almost daily, 1–4 times a week, 1–3 times a month, never or special occasions only), education (college or university degree, high school, below), physical activity (MET-min/week, continuous), history of hypertension, cardiovascular disease, diabetes (yes, no), and cardiometabolic biomarkers (triglycerides, LDL-c, hs-CRP).

Model 5 was adjusted for age (years, continuous), sex (male, female), race (White, Asian, Black, Mixed), BMI (kg/m^2^, continuous), Townsend deprivation index (continuous), smoking status (never, previous, current), alcohol consumption (daily or almost daily, 1–4 times a week, 1–3 times a month, never or special occasions only), education (college or university degree, high school, below), physical activity (MET-min/week, continuous), history of hypertension, cardiovascular disease, diabetes (yes, no), and eGFR.

**^a^** Test for trend based on variables containing the median value for each quartile.

*Abbreviations:* CIs, confidence intervals; CKD, chronic kidney disease; HRs, hazard ratios; eGFR, estimated glomerular filtration rate; LDL-C, low-density lipoprotein cholesterol; hs-CRP, high-sensitive C-reactive protein; SD, standard deviation.

**Supplementary Table S8. Associations (HRs and 95% CIs) of KDM-BA acceleration and PhenoAge acceleration with incident CKD excluding participants with pre-existing cardiovascular disease and diabetes (n = 257,228).**

| **Biological ages** | **Cases (n, %)** | **Model 1** | **Model 2** | **Model 3** |
| --- | --- | --- | --- | --- |
|  |  | **HR (95% CI)** | **HR (95% CI)** | **HR (95% CI)** |
| **KDM-BA acceleration (Quartiles)** | | | | |
| Q1 | 1,138 (1.77%) | 1.00 (Ref) | 1.00 (Ref) | 1.00 (Ref) |
| Q2 | 1,254 (1.95%) | 1.09 (1.01, 1.18) | 1.25 (1.16, 1.36) | 1.22 (1.13, 1.33) |
| Q3 | 1,284 (2.00%) | 1.11 (1.02, 1.20) | 1.43 (1.30, 1.56) | 1.38 (1.26, 1.51) |
| Q4 | 1,956 (3.04%) | 1.71 (1.59, 1.83) | 2.02 (1.84, 2.21) | 1.88 (1.71, 2.05) |
| *P*-trend^a^ |  | <0.0001 | <0.0001 | <0.0001 |
| **Continuous (per SD increment)** |  | 1.26 (1.22, 1.29) | 1.35 (1.31, 1.40) | 1.32 (1.27, 1.36) |
| **PhenoAge acceleration (Quartiles)** | | | | |
| Q1 | 664 (1.03%) | 1.00 (Ref) | 1.00 (Ref) | 1.00 (Ref) |
| Q2 | 1,050 (1.63%) | 1.59 (1.45, 1.76) | 1.42 (1.29, 1.57) | 1.42 (1.28, 1.56) |
| Q3 | 1,511 (2.35%) | 2.31 (2.11, 2.53) | 1.88 (1.71, 2.06) | 1.86 (1.70, 2.04) |
| Q4 | 2,407 (3.74%) | 3.77 (3.46, 4.10) | 2.81 (2.57, 3.08) | 2.77 (2.53, 3.03) |
| *P*-trend^a^ |  | <0.0001 | <0.0001 | <0.0001 |
| **Continuous (per SD increment)** |  | 1.51 (1.48, 1.54) | 1.43 (1.40, 1.46) | 1.43 (1.40, 1.46) |

*****HRs and 95% CIs were calculated with the use of the cause-specific competing risk model.

Model 1 was not adjusted.

Model 2: adjusted for age (years, continuous), sex (male, female), race (White, Asian, Black, Mixed), BMI (kg/m^2^, continuous), Townsend deprivation index (continuous), smoking status (never, previous, current), alcohol consumption (daily or almost daily, 1–4 times a week, 1–3 times a month, never or special occasions only), education (college or university degree, high school, below), and physical activity (MET-min/week, continuous).

Model 3: Model 2 + history of hypertension (yes, no).

**^a^** Test for trend based on variables containing the median value for each quartile.

*Abbreviations:* CIs, confidence intervals; CKD, chronic kidney disease; HRs, hazard ratios; SD, standard deviation.
